# Counterfactual thinking induces different neural patterns of memory modification in anxious individuals

**DOI:** 10.1038/s41598-024-61545-x

**Published:** 2024-05-09

**Authors:** Shenyang Huang, Leonard Faul, Natasha Parikh, Kevin S. LaBar, Felipe De Brigard

**Affiliations:** 1 Department of Psychology and Neuroscience, Duke University, Durham, NC 27708 USA; 2 Center for Cognitive Neuroscience, Duke University, Durham, NC 27708 USA; 3 Department of Psychology and Neuroscience, Boston College, Chestnut Hill, MA 02467 USA; 4 Department of Psychology and Neuroscience, University of North Carolina at Chapel Hill, Chapel Hill, NC 27599 USA; 5 Department of Philosophy, Duke University, Durham, NC 27708 USA

**Keywords:** Emotion, Long-term memory, Human behaviour

## Abstract

Episodic counterfactual thinking (eCFT) is the process of mentally simulating alternate versions of experiences, which confers new phenomenological properties to the original memory and may be a useful therapeutic target for trait anxiety. However, it remains unclear how the neural representations of a memory change during eCFT. We hypothesized that eCFT-induced memory modification is associated with changes to the neural pattern of a memory primarily within the default mode network, moderated by dispositional anxiety levels. We tested this proposal by examining the representational dynamics of eCFT for 39 participants varying in trait anxiety. During eCFT, lateral parietal regions showed progressively more distinct activity patterns, whereas medial frontal neural activity patterns became more similar to those of the original memory. Neural pattern similarity in many default mode network regions was moderated by trait anxiety, where highly anxious individuals exhibited more generalized representations for upward eCFT (better counterfactual outcomes), but more distinct representations for downward eCFT (worse counterfactual outcomes). Our findings illustrate the efficacy of examining eCFT-based memory modification via neural pattern similarity, as well as the intricate interplay between trait anxiety and eCFT generation.

## Introduction

Have you ever found yourself pondering over a past event and imagining how it could have occurred differently? Perhaps you got stuck in traffic and missed an important meeting, leaving you wondering whether you could have made it in time, had you picked a different route. This act of mentally simulating ways in which past personal experiences could have occurred differently is known as episodic counterfactual thinking (episodic CFT or eCFT)^1^. Sometimes we engage in eCFT spontaneously, such as during moments of mind-wandering when we reminisce about the past and consider alternate possibilities^2^. One can also engage in eCFT intentionally during goal-oriented attempts at emotion regulation, which can alter how past experiences are perceived^3,4^. Specifically, *downward* eCFT (simulating a counterfactual experience *worse* than the true outcome, e.g., “at least…”) typically produces feelings of contentment or relief that consequently mollifies emotional elements of the original memory; in contrast, *upward* eCFT (simulating a counterfactual experience *better* than the true outcome, e.g., “if only…”) typically produces feelings of regret or guilt^5,6^. Both downward and upward eCFT can be adaptive processes that either remind us that experiences are not as bad as they could have been or motivate us to adjust future behaviors in order to achieve more desirable outcomes. A growing body of work has sought to investigate how engaging in such mental simulations modifies the phenomenology of remembering the original event later on. One study, for instance, found differences in the perceived valence and detail of participants’ own autobiographical memories before and after upward or downward eCFT^7^. Others have observed that engaging in downward eCFT reduces the regret and arousal associated with negative autobiographical memories^8^; relatedly, using positive reinterpretation of negative memories is found to enhance positive emotions and positive content in subsequent recollections^9^. Moreover, accumulating evidence that eCFT significantly shapes our recollection of autobiographical experiences has garnered interest in understanding the role that aberrant eCFT patterns may play in maintaining psychological disorders, as well as the utility of eCFT-based therapeutic interventions^1^.

Indeed, eCFT can turn maladaptive when ruminating on unattained outcomes conjures excessive disappointment and regret, or when brooding on hypothetically worse situations impedes disengagement from pathologically prolonged negative mood states^10,11^. Aberrant engagement with eCFT is implicated in many psychiatric and neurological disorders, including anxiety, depression, schizophrenia, and post-traumatic stress disorder^12^. Importantly, eCFT also appears to be impacted by individual differences in dispositional traits among nonclinical samples. For instance, prior work has shown that individuals who report experiencing high anxiety also report engaging in eCFT more frequently and perceive eCFT to be a more negative experience^13^. Highly anxious individuals tend to describe their counterfactual simulations (especially upward eCFT of negative events) with less detail and perceive them as less likely to have occurred relative to individuals with lower levels of anxiety^14^. Moreover, trait anxiety moderates eCFT-induced changes in the phenomenology of recalling the original event, such that more anxious individuals experience a greater reduction in emotional arousal and regret of their negative personal events after engaging in downward eCFT as a regulation strategy^8^. This latter finding demonstrates that targeted implementation of eCFT can aid in mollifying feelings of regret in anxious individuals.

These eCFT-induced changes in memory phenomenology are theorized to emerge via *reconsolidation*: recalling a past event makes the memory trace susceptible to modification, and engaging in eCFT may integrate new details and feelings associated with the mental simulation into the original memory content, resulting in an altered memory trace^15,16^. Supporting this reconsolidation proposal, fMRI studies have found that engaging in eCFT recruits regions in the default mode network (DMN), which are commonly implicated in autobiographical memory recall^17,18^. In fact, activation in the medial prefrontal cortex (PFC), angular gyrus (AG), and superior temporal gyrus have consistently been shown to be even more active during episodic counterfactual simulation than episodic recall^18^. This enhanced activity is thought to reflect the added demands placed upon this neural system during the extraction and recombination of episodic elements from past experiences to construct a mental simulation of a hypothetical event. Indeed, eCFT entails not only extracting elements from memory but also introducing novel modifications, both of which implicate DMN regions^19^. Closer inspections of different mental simulations have revealed that DMN regions are differentially engaged depending on what aspects of memory are manipulated. For instance, studies have shown divergent brain activations recruited by episodic vs. semantic CFT^20^, eCFT vs. visual perspective shifts^21^, person- vs. object-based eCFT^22^, and action- vs. situation-based eCFT^23^. Collectively, these findings suggest the nuanced nature of eCFT and its neural underpinnings.

To date, however, fMRI studies of eCFT have largely focused on *univariate activation*—averaged magnitude of neural activity in a cluster of voxels—during episodic memory recall and episodic counterfactual simulation. An alternative strategy is to examine the *multi-voxel activity pattern* that may represent the content of memory or simulation, providing a more direct and sensitive test of memory modification. One such analytical technique is representational similarity analysis^24^, which first computes the (dis)similarity of activity patterns across events, or *neural pattern similarity (NPS)*, and then examines these NPS values as a function of behavioral measures or experimental conditions. Taking this approach, one study examined activity patterns in the hippocampus and ventral striatum on repeated recollections of the same negative memories, finding that lower same-memory NPS in those regions was correlated with greater positive change in the phenomenology of recall^9^. In other words, this neural index of changes in activity patterns provided information on modified subjective experiences and, likely, memory content. However, this work studied positive memory reinterpretations more broadly rather than eCFT, thus not assessing how neural patterns shifted during the initial act of simulating hypothetical events based on memory. Additional work is needed to precisely discern brain regions whose activity pattern changes as autobiographical memories are freshly modified and reconsolidated via eCFT. Researchers can leverage this understanding to not only gain a richer appreciation for the DMN’s role in supporting eCFT but also identify more specific neural targets for therapeutic interventions that seek to amplify emotion regulation and modification of autobiographical memories.

**Figure 1:**
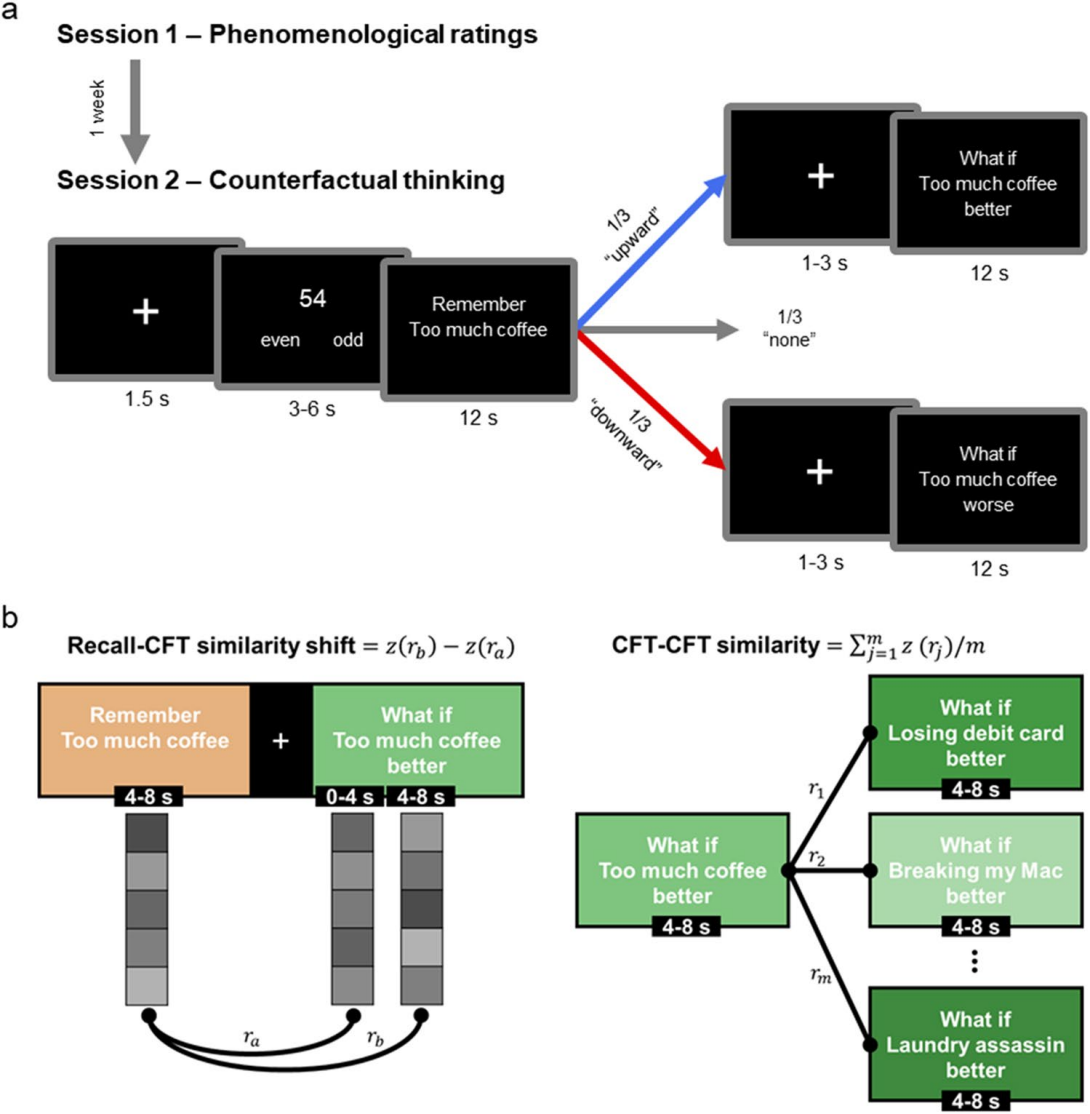
Experimental paradigm and analytical approach. **(a)** In Session 1, participants described 45 negative, regretful personal events that occurred within the past 10 years by generating a short title (e.g., “Too much coffee”) and providing phenomenological ratings for each memory. In Session 2, participants were cued with titles to recall their autobiographical memories; after recalling each event, participants were instructed to perform one of the following: (1) simulate a worse counterfactual outcome, (2) simulate a better counterfactual outcome, or (3) move on to the next trial without explicit mental simulations. **(b)** We computed two trial-level neural pattern similarity (NPS) indices. The Recall-CFT similarity shift index (left) is computed for each trial as the change in the NPS value (Fisher z-transformed correlations) between Recall and CFT patterns across two timepoints of counterfactual simulation. Effects of the jittered interval between Recall and CFT (1–3 s) and the condition of the previous trial were estimated in a regression model and removed from the raw index. The CFT–CFT similarity index (right) is computed for each trial as the average of NPS values between a given CFT pattern and all other same-condition CFT patterns. See Supplementary Fig. S1c for phenomenological ratings of the memories, as well as average recall and CFT generation times.

We aimed to fill this gap in the literature by assessing how the neural activity pattern representing autobiographical memory changes over time as participants simulate better or worse counterfactual outcomes. To this end, we designed a multi-session experiment in which participants recalled negative autobiographical memories and engaged in both upward and downward eCFT (see Fig. 1a). Specifically, in Session 1, participants recalled and described negative, regretful personal events and rated the phenomenological characteristics of their memories (e.g., valence, arousal, detail). For each event, participants generated a short title (e.g., “Too much coffee”) to be used as the subsequent retrieval cue. One week later, at Session 2, participants were cued to recall their own memories while undergoing MRI scanning. In particular, after each recall, participants were randomly instructed to perform one of the following: (1) simulate a worse counterfactual outcome (downward eCFT), (2) simulate a better counterfactual outcome (upward eCFT), or (3) move on to the next trial without explicitly engaging in eCFT (No eCFT). In line with the *constructive episodic simulation hypothesis*^19^, we hypothesized that regions within the DMN would exhibit increased univariate activation during eCFT compared to autobiographical recall. Crucially, with this experimental design and a multi-voxel pattern similarity analysis approach, we computed two event-specific neural indices for our multivariate analysis: Recall-CFT similarity shift index and CFT-CFT similarity index (see Fig. 1b). The Recall-CFT similarity shift index measures for each memory the extent to which eCFT-induced memory modification increases or decreases over time, whereas the CFT-CFT similarity index (computed separately for upward and downward eCFT) measures the specificity of a simulated counterfactual content in relation to all other simulations of the same kind.

According to the cortical binding of relational activity (CoBRA) theory, the ventral posterior parietal cortex acts as a convergence zone for binding episodic event features in memory^25^. In particular, the AG has been regarded as a crucial hub region for constructive episodic processes such as future thinking^26^. Thus, we expected a consistently negative Recall-CFT similarity shift index from this region, which would indicate the active process of mentally manipulating, updating, and recombining existing mnemonic elements during eCFT as neural patterns become more dissimilar from the original memory. In contrast, since the episodic counterfactual simulations need to stem from some remembered episode, other DMN regions may need to maintain the original autobiographical memory during eCFT, or facilitate similar mental operations involving scene construction, emotional appraisal, and self-reflection. For instance, the medial PFC has been suggested to coordinate hippocampal-dependent processes that orchestrate autobiographical memory recall^27^ while also representing the emotional intensity and personal significance of emotional stimuli^28^. Such regions maintaining pre-existing episodic components as the foundational elements for counterfactual simulations are expected to have a consistently positive Recall-CFT similarity shift index, given that the original pattern of the memory is recapitulated. Thus, our multivariate NPS analysis aimed to identify brain regions reflecting both the updating and possible maintenance of episodic content, which we expected to arise from different regions of the DMN.

Given past behavioral evidence that trait anxiety modulates both the phenomenology of eCFT^14^ and the effect of eCFT on subsequent memory recall^8^, we further hypothesized that our neural indices of memory modification and counterfactual specificity would be sensitive to an individual’s dispositional anxiety. To examine these effects, we recruited participants across a spectrum of trait anxiety levels (see Supplementary Fig. S1a). With limited research in this area, however, we hypothesized two possible associations between anxiety and neural patterns during counterfactual generation. First, individuals with higher social anxiety have been shown to generate more upward counterfactuals for imagined scenarios^29,30^, which may suggest that anxious individuals generate more specific mental simulations for hypothetical better outcomes that are also more distinct in their neural representations than hypothetical worse outcomes. However, whether such effects generalize to eCFT of personal autobiographical memories is unclear. A greater propensity for generating upward counterfactuals, on the other hand, may not necessarily be associated with more distinct neural representations. As mentioned, higher anxiety is associated with less detail for upward counterfactuals, suggesting that upward counterfactuals may instead be associated with more generalized neural representations than downward counterfactuals^14^. Such effects may be explained by negative biases among anxious individuals that engender greater difficulty perceiving positive alternatives as likely to occur^14^, and more expectations for negative future events^31^. Therefore, using our novel analytical approach that examines the representational dynamics of eCFT, we examined which of these hypothesized associations would be supported by our data.

## Results

### Univariate analysis

**Figure 2:**
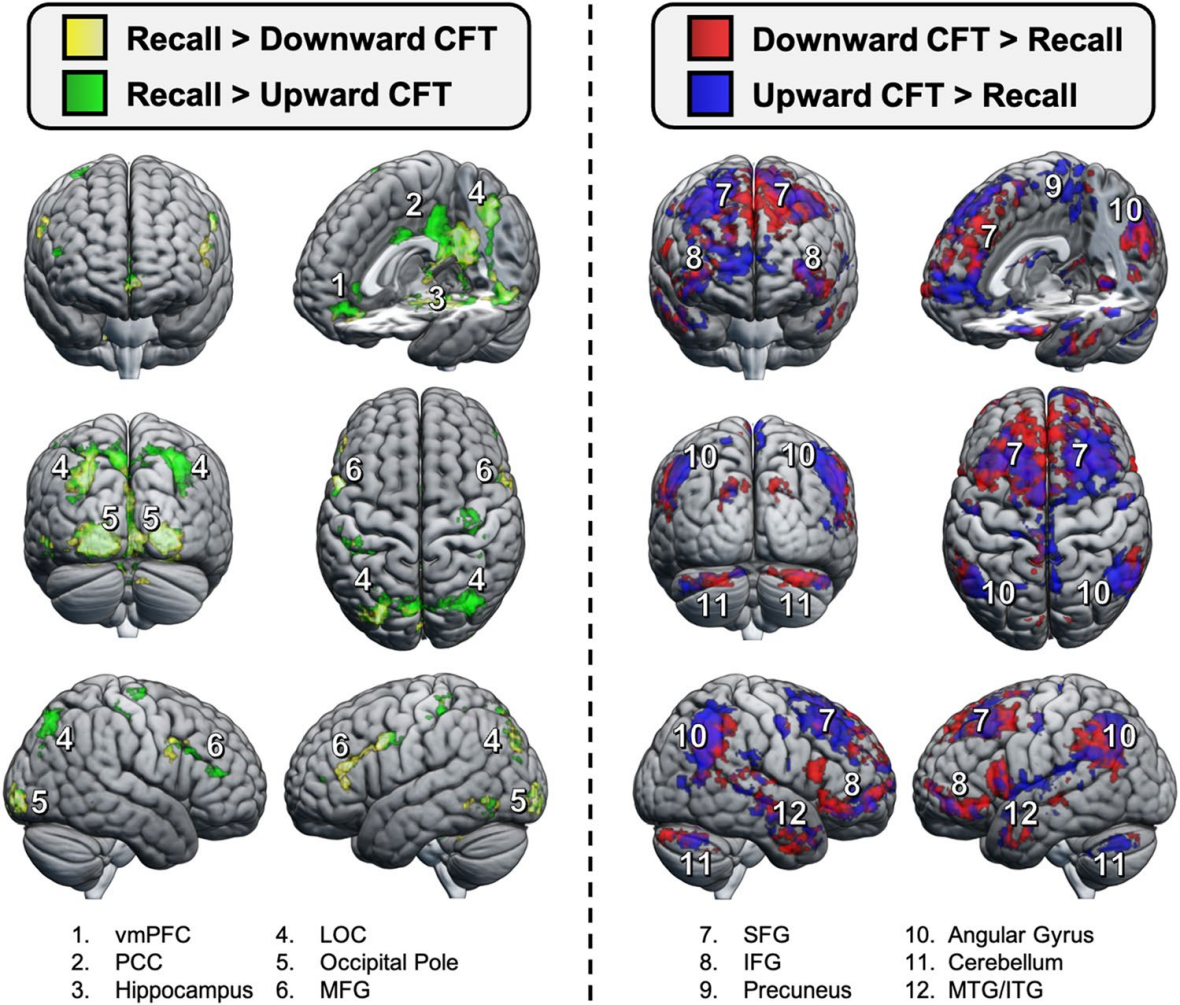
Whole-brain univariate analyses. Effects are shown for regions more active during natural recall compared to eCFT (left), as well as regions more active during eCFT compared to natural recall (right) during the full 12 s of recall/simulation. The 3D rendered brains represent the group results after mixed effects analysis (z  > 2.3, cluster-corrected p  < .05). Note that the same preprocessed data was used for both univariate and multivariate assessments, without smoothing. These contrasts reveal that naturally recalling autobiographical memories as they occurred elicited greater activation primarily in occipital and hippocampal/parahippocampal regions, whereas generating counterfactual outcomes elicited greater activation in medial/lateral prefrontal regions, lateral parietal and temporal cortices, and the cerebellum. See Supplementary Fig. S2 and Supplementary Table S1 for a full overview of activation differences between conditions, as well as main effects. *vmPFC* ventromedial prefrontal cortex, *PCC* posterior cingulate cortex, *LOC* lateral occipital cortex, *MFG* middle frontal gyrus, *SFG* superior frontal gyrus, *IFG* inferior frontal gyrus, *MTG* middle temporal gyrus, *ITG* inferior temporal gyrus.

Supporting previous neuroimaging studies on autobiographical recall and eCFT, all conditions (Recall, upward eCFT, and downward eCFT) recruited activation predominantly in the DMN encompassing regions such as the dorsomedial PFC, lateral parietal and temporal cortices, hippocampus, and cerebellum (for individual 2D slices, see Supplementary Fig. S2). To identify regions that were differentially activated during eCFT compared to recall, we examined the contrasts [Upward CFT > Recall] and [Downward CFT > Recall], as well as the reverse contrasts (see Fig. 2; for individual 2D slices, see Supplementary Fig. S2). These analyses revealed that engaging in eCFT elicited *greater* activation in dorsomedial, dorsolateral, and ventrolateral PFC (including the inferior and superior frontal gyri, as well as frontal pole), AG, lingual gyrus, inferior/middle temporal gyrus, caudate, and cerebellum when compared to natural memory recall (see Supplementary Table S1). Conversely, eCFT exhibited *reduced* activation predominantly in hippocampal/parahippocampal regions, occipital cortex, posterior cingulate cortex, and ventromedial PFC.

To identify regions most sensitive to eCFT processes as the focus of subsequent multivariate neural pattern similarity analyses, we performed an F-test across all four contrasts ([Downward CFT > Recall], [Recall > Downward CFT], [Upward CFT > Recall], and [Recall > Upward CFT]), thereby isolating regions that exhibited either an increase or decrease in activation when participants constructed counterfactual outcomes. We retained ROIs from the Automated Anatomical Labelling atlas 3 for which clusters from the F-test covered at least 25% of the voxels (see Methods). This approach identified 56 primary ROIs that we further examined with respect to their neural pattern similarity (see Supplementary Table S2 for a list of all ROIs and the percent voxel overlap between the ROI size and the observed univariate clusters).

### Neural pattern similarity

#### Change over time in neural pattern similarity from autobiographical memory recall to CFT simulations (recall-CFT similarity shift index)

We first examined the similarity between the neural pattern of the original autobiographical event and that of the associated episodic counterfactual simulation. Importantly, whereas our univariate analyses identified regions that differed in the *magnitude* of activation between eCFT and natural recall, this multivariate analysis identified which regions were changing in their neural signature—or pattern of activation—during counterfactual generation. Given that we calculated the change in recall-CFT similarity from early (0–4 s) to late (4–8 s) temporal epochs while participants engaged in episodic counterfactual simulation (i.e., the similarity shift index), we interpret a decrease in similarity as indicative of the neural representational pattern for the simulated counterfactual content becoming more different from the original memory. Accordingly, our analyses revealed that Recall-CFT similarity in a set of both left and right parietal regions *reduced* as participants elaborated on the simulated counterfactual content (left SMG, b = −0.048, SE = 0.015, t(57.7) =  −3.12, P = 0.003, P_FDR_ = 0.023; left AG, b = −0.045, SE = 0.014, t(59.1) =  −3.12, P = 0.003, P_FDR_ = 0.023; and right AG, b = −0.059, SE = 0.016, t(52.8) =  −3.77, P < 0.001, P_FDR_ = 0.008; see Supplementary Table S3). These effects were irrespective of the type of eCFT that was generated (all Condition main effects and interactions were P_FDR_ > 0.05).

**Figure 3:**
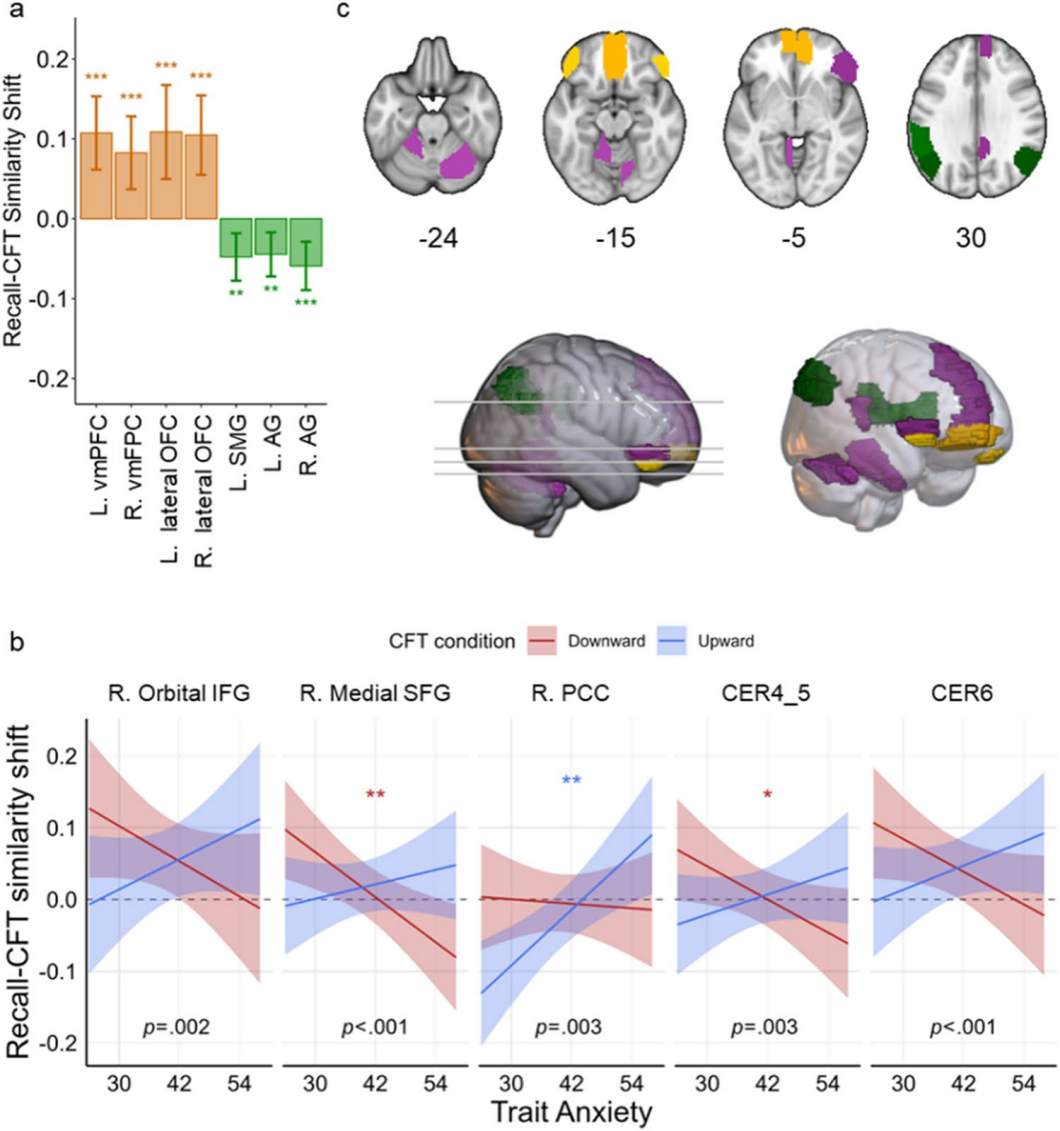
Recall-CFT similarity shift analysis. Recall-CFT similarity shift index (difference of Fisher z-transformed correlation coefficients) in each brain region was regressed on eCFT condition (upward vs. downward) and participant trait anxiety. Positive values indicate increasing correspondence between neural patterns of recalled and simulated contents, while negative values indicate decreasing correspondence. **(a)** Columns illustrate fitted intercepts of the Recall-CFT similarity shift index for brain regions whose intercepts are significantly different from zero. **(b)** Regression plots illustrate significant trait-anxiety-by-CFT-condition interactions on Recall-CFT similarity shift index for different brain regions. Error bars in (**a**) and shaded bands in (**b**) indicate 95% confidence intervals. The significance of all effects was determined based on P-values corrected for False Discovery Rate (FDR; Q < 0.05). Uncorrected P-values for the interaction (slope difference) are reported in text, and significance levels for the intercepts in **(a)** and simple slopes in **(b)** are annotated with asterisks, *P < 0.05; **P < 0.01; ***P < 0.001. **(c)** Brain regions demonstrating memory maintenance (yellow), counterfactual elaboration (green), and significant trait-anxiety-by-CFT-condition interaction (purple). *vmPFC* ventromedial prefrontal cortex, *OFC* orbital frontal cortex, *SMG* supramarginal gyrus, *AG* angular gyrus, *IFG* inferior frontal gyrus, *SFG* superior frontal gyrus, *PCC* posterior cingulate cortex, *CER4_5* lobule IV, V of cerebellar hemisphere, *CER6* lobule VI of cerebellar hemisphere. See also Supplementary Fig. S3.

Interestingly, some regions exhibited an *increase* in Recall-CFT similarity as participants generated counterfactual outcomes, which may be associated with recreating the original neural representational pattern from initial memory recall, but may also support reengagement of similar episodic processes. Specifically, this increase in similarity was primarily confined to a set of ventral frontal regions (left vmPFC, b = 0.107, SE = 0.024, t(50.7) = 4.56, P < 0.001, P_FDR_ = 0.002; right vmPFC, b = 0.083, SE = 0.023, t(50.7) = 3.52, P = 0.001, P_FDR_ = 0.010; left lateral OFC, b = 0.109, SE = 0.030, t(49.7) = 3.59, P = 0.001, P_FDR_ = 0.010; and right lateral OFC, b = 0.105, SE = 0.026, t(54.1) = 4.09, P < 0.001, P_FDR_ = 0.004; see Fig. 3, Supplementary Fig. S3, and Supplementary Table S3). Again, these effects were irrespective of the type of eCFT that was generated (all Condition main effects and interactions were P_FDR_ > 0.05).

Although these decreases and increases in Recall-CFT similarity were not specific to the type of the eCFT generated, we suspected that trait anxiety may play a moderating role in how neural patterns shifted during eCFT. Indeed, our analyses revealed a significant interaction between eCFT condition and trait anxiety in several regions in the frontal lobe (right orbital IFG, b = 0.008, SE = 0.003, t(1006.7) = 3.04, P = 0.002, P_FDR_ = 0.039; right medial SFG, b = 0.007, SE = 0.002, t(1008.3) = 3.46, P = 0.001, P_FDR_ = 0.024; left vmPFC, b = 0.008, SE = 0.003, t(1008.5) = 2.79, P = 0.005, P_FDR_ = 0.050; right PCC, b = 0.07, SE = 0.002, t(1010.7) = 2.93, P = 0.003, P_FDR_ = 0.039) and in the cerebellum (CER4_5, b = 0.006, SE = 0.002, t(1009.2) = 2.99, P = 0.003, P_FDR_ = 0.039; CER6, b = 0.007, SE = 0.002, t(1007.4) = 3.34, P = 0.001, P_FDR_ = 0.024). Notably in all these regions, the significant interaction was due to participants with higher trait anxiety levels, relative to participants with lower anxiety, having elaborated *better* counterfactual content more similar to the original event but having elaborated *worse* counterfactual content more distinct from the original event. In other words, within these regions, increased trait anxiety was associated with greater recapitulation of the original neural signature when asked to generate better counterfactual outcomes, while generating worse counterfactual outcomes were typically associated with more change of the neural signature. Speculatively, such a pattern of results suggests that more anxious individuals are more capable of vividly simulating detailed alternative scenarios in which things could have been worse (downward eCFT) but more generalized scenarios in which things could have been better (upward eCFT).

#### Similarity among counterfactual simulations within the same condition (CFT–CFT similarity index) is also associated with trait anxiety

Thus far, our analyses indicate that engaging in eCFT elicits both increases (DMN) and decreases (occipito-temporal) in activation compared to naturally recalling an autobiographical memory. However, these regions further dissociate in how their neural patterns shift during eCFT generation, with (1) lateral parietal regions exhibiting a decrease in similarity, (2) inferior frontal regions exhibiting an increase in similarity, and (3) frontal, parietal, and cerebellar regions uniquely shifting in NPS depending on the type of eCFT that is simulated and individual differences in trait anxiety. This third effect was particularly compelling in suggesting that highly anxious individuals generate better counterfactual outcomes whose neural signatures are less distinct from the original memory.

**Figure 4:**
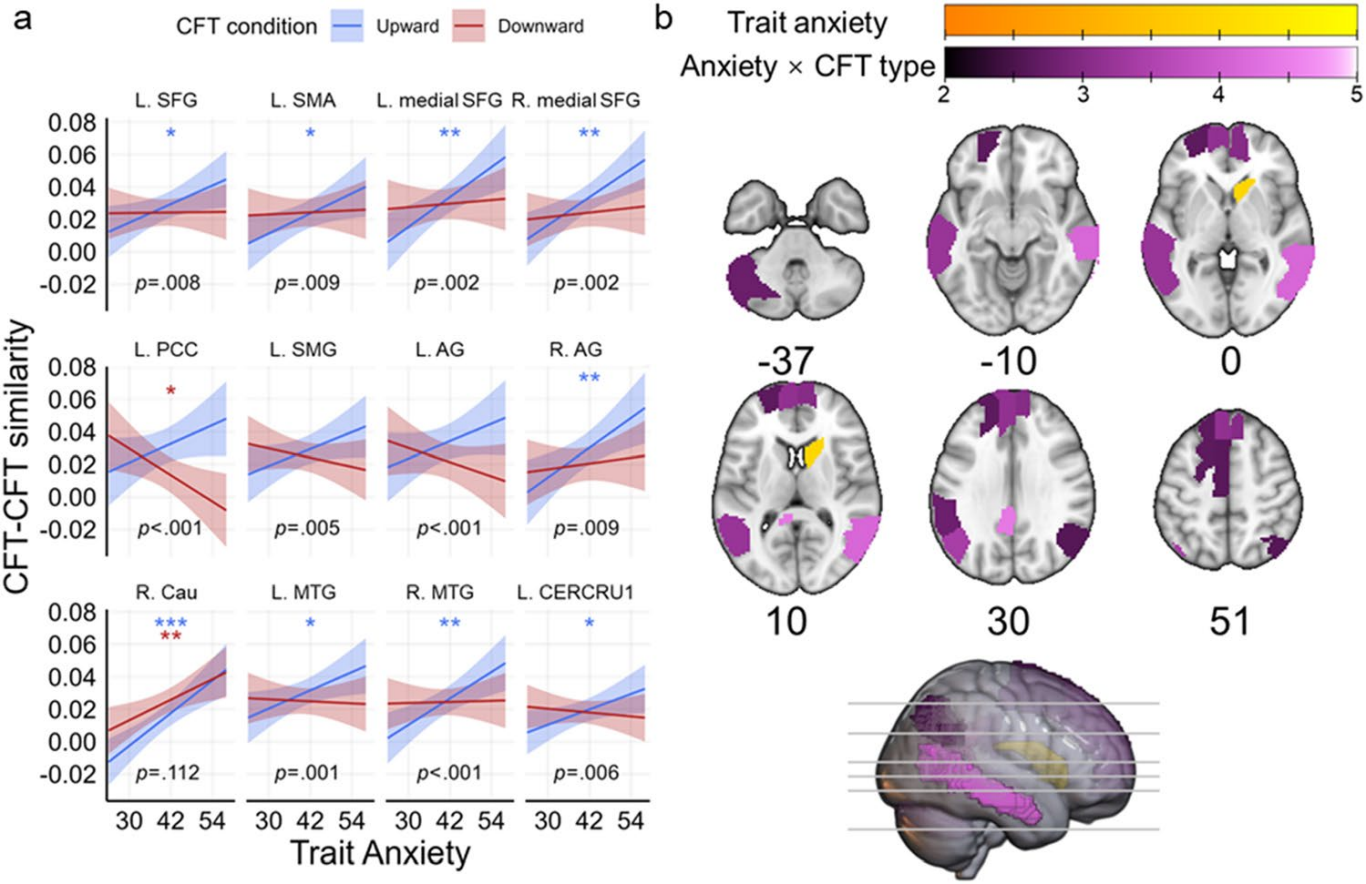
CFT–CFT similarity analysis. (**a**) Regression lines of different brain regions’ CFT-CFT similarity (Fisher z-transformed correlation coefficients) on participant trait anxiety. Higher values indicate more generalized patterns among counterfactual outcomes in the same condition (upward eCFT or downward eCFT), whereas lower values reflect greater differentiation. Plots depict estimated marginal means and shaded bands indicate 95% confidence intervals. Uncorrected *p*-values for the trait-anxiety-by-CFT-condition interaction (slope difference) are reported in text, and significance levels for simple slopes are annotated with asterisks, *P < 0.05; **P < 0.01; ***P < 0.001. **(b)** Brain regions showing a significant main effect (yellow) or trait-anxiety-by-CFT-condition interaction (purple). Color intensity based on the *t*-stat of the respective effect. *SFG* superior frontal gyrus, *SMA* supplementary motor area, *PCC* posterior cingulate cortex, *SMG* supramarginal gyrus, *AG* angular gyrus, *Cau* caudate nucleus, *MTG* middle temporal gyrus, *CERCRU1* Crus I of cerebellar hemisphere. See also Supplementary Fig. S4.

To investigate this effect further, we next compared eCFT within each condition (upward or downward), as we hypothesized that highly anxious individuals would generate better counterfactual outcomes that are more generalized in their neural representations. Regions that exhibit higher similarity across upward and downward eCFT are indicative of a more common neural signature, whereas regions that display lower similarity across eCFT in the same condition are indicative of more distinct neural signatures. Again, we also tested the effect of trait anxiety on these similarity indices. Indeed, across all ROIs tested, the right caudate nucleus showed a significant main effect of trait anxiety on CFT-CFT similarity (b = 0.013, SE = 0.003, t(36) = 3.96, P < 0.001, P_FDR_ = 0.019), indicating that increased anxiety is associated with more common signatures of both upward and downward CFT within the caudate nucleus (see Fig. 4, Supplementary Fig. S4, and Supplementary Table S4).

Critically, a distributed group of brain regions exhibited significant interactions between trait anxiety and eCFT condition (left SFG: b = 0.009, SE = 0.003, t(1013.4) = 2.66, P = 0.008, P_FDR_ = 0.048; left SMA: b = 0.009, SE = 0.003, t(1012.9) = 2.62, P = 0.009, P_FDR_ = 0.048; left medial SFG: b = 0.013, SE = 0.004, t(1013.4) = 3.15, P = 0.002, P_FDR_ = 0.019; right medial SFG: b = 0.011, SE = 0.004, t(1014.1) = 3.04, P = 0.002, P_FDR_ = 0.022; left PCC: b = 0.022, SE = 0.005, t(1014.0) = 4.42, P < 0.001, P_FDR_ = 0.001; left SMG: b = 0.013, SE = 0.005, t(1014.9) = 2.79, P = 0.005, P_FDR_ = 0.042; left AG: b = 0.015, SE = 0.005, t(1013.0) = 3.41, P = 0.001, P_FDR_ = 0.013; right AG: b = 0.012, SE = 0.004, t(1013.5) = 2.60, P = 0.009, P_FDR_ = 0.048; left MTG: b = 0.010, SE = 0.003, t(1012.7) = 3.23, P = 0.001, P_FDR_ = 0.018; right MTG: b = 0.012, SE = 0.003, t(1012.7) = 4.14, P < 0.001, P_FDR_ = 0.001; left CERCRU1: b = 0.009, SE = 0.003, t(1013.7) = 2.76, P = 0.006, P_FDR_ = 0.042). Noticeably, in all these regions the significant interactions were driven by a more positive association between trait anxiety and CFT-CFT similarity for upward eCFT than for downward eCFT. In other words, highly anxious individuals tended to simulate better counterfactual outcomes that were more generalized in their neural representations (i.e., similar to each other); in contrast, worse counterfactuals were relatively more differentiated (i.e., less similar to each other).

## Discussion

This research examined how multivariate neural patterns associated with specific autobiographical memories change during directed eCFT. Our univariate findings support the constructive episodic simulation hypothesis by demonstrating greater activation in core DMN regions during eCFT compared to naturally recalling an autobiographical memory^17,19^. Specifically, when participants generated counterfactual alternatives to their autobiographical memories, activation primarily decreased in visual and hippocampal regions, but increased throughout core DMN regions located in prefrontal, parietal, and temporal cortices, as well as within the cerebellum, likely owing to greater demands for mental construction and episodic binding during the simulation process^25^. Our multivariate NPS analysis further expands upon these findings by identifying neural patterns in the parietal cortex that decrease in similarity during eCFT when compared to the original memory, while the ventral PFC exhibits neural patterns that increase in similarity. Additional analyses that examined the moderating role of trait anxiety on Recall-CFT NPS as well as CFT-CFT NPS revealed that highly anxious individuals exhibit neural patterns of activation that are more generalized for better counterfactual alternatives (upward eCFT) and more differentiated for worse counterfactual alternatives (downward eCFT). In what follows, we discuss each of these NPS effects and their implications.

### Neural similarity between autobiographical memory recall and CFT decreases over time in posterior parietal cortex and increases over time in ventral prefrontal cortex

Our analyses of Recall-CFT similarity revealed that bilateral AG and left SMG decreased in similarity as participants generated both better and worse counterfactual outcomes. While our univariate assessment indicated that these parietal regions were generally more active during eCFT than initially recalling the memory, our multivariate assessment further showed that their activation signature also became more dissimilar from the original memory during counterfactual construction. In line with the constructive episodic simulation hypothesis, these regions may be exhibiting a different neural pattern due to the differential demands placed upon the episodic memory system when flexibly combining episodic features to mentally construct and simulate a novel, hypothetical event—as opposed to merely reconstructing an actual, experienced event. We also interpret these findings as consistent with the CoBRA theory as they show that the posterior parietal cortex is particularly involved in facilitating the binding of episodic features^25^, owing to the fact that it was one of the few regions identified in our NPS analysis as changing during eCFT. This result also converges with the Contextual Integration Model’s proposal, according to which multimodal event details are specifically integrated within the AG^26^. Our findings do, however, suggest that the neural signatures underlying memory reconstruction are different from those supporting counterfactual simulation. That is, activation in the AG seems to be sensitive to *how* event details are combined to simulate an episodic event, given its proposed role as a convergence zone for integrating event features from disparate cortical regions^25^.

We therefore interpret decreasing Recall-CFT NPS in the posterior parietal cortex in the present study as demonstrative of autobiographical memories being modified–and potentially reconsolidated–via counterfactual thought, such that neural patterns representing the original memory became more dissimilar as the memory was being actively manipulated. This interpretation is further supported by causal evidence specifically linking the AG to mental simulation processes. One study asked participants to perform simulation (future thinking), divergent thinking (creative uses), and nonepisodic control tasks after receiving inhibitory continuous theta-burst stimulation (cTBS) to the AG or the vertex^32^. Compared to vertex stimulation, AG stimulation was associated with fewer internal details reported for the simulation task, as well as reduced fluency and flexibility on the divergent thinking task, while no differences were found for the nonepisodic control task. Moreover, cTBS to the AG versus vertex resulted in reduced hippocampal activity in the episodic simulation and divergent thinking tasks, but not the nonepisodic control task. These findings illustrate that the AG facilitates downstream effects on hippocampal activity to influence episodic processes, and thus emphasizes the causal role that the AG plays in producing detailed simulations of hypothetical events^32^. Importantly, our present findings provide novel evidence that specifically links shifting neural patterns in the AG with counterfactual modification of autobiographical memories in real time. We note, however, that more research is needed to confirm whether changing NPS in the posterior parietal cortex actually alters the original memory trace, as well as whether decreased Recall-CFT NPS in this region might represent other processes that are uniquely engaged during counterfactual thinking compared to initial memory recall, such as shifts in visual perspective that may naturally accompany mental modifications of a memory and are known to also recruit this area of the brain^33^.

In contrast to this decreasing NPS effect, the bilateral vmPFC and lateral OFC were instead associated with Recall-CFT neural patterns becoming *more similar* over time during eCFT. Here, again, it is important to note that we specifically evaluated a *change* in similarity as participants engaged in their counterfactual simulations. As such, our results do not suggest that bilateral vmPFC and lateral OFC were the only regions that exhibited similar neural patterns between autobiographical recall and eCFT, but that these regions specifically *increased* in neural pattern similarity as the counterfactual was generated. One possible interpretation is that this increase in similarity is indicative of the original neural signature of the autobiographical memory recapitulating during eCFT, perhaps serving to maintain key aspects of the event that are not modified. That is, generating a counterfactual alternative of a negative autobiographical memory (e.g., being hit by a car while riding a bike) requires both maintenance of the core features of an episodic event (e.g., riding a bike), as well as manipulating specific aspects to produce the imagined alternative outcome (e.g., stopping and looking before crossing the intersection). Note, however, that what is maintained versus manipulated can vary considerably across individuals and memories. Another possibility, though, is that these prefrontal regions are supporting similar processes that unfold during both recall and eCFT, but that these processes are not specific to the episodic details being manipulated. For instance, prior work has demonstrated a critical role for the vmPFC in scene construction^34,35^. Neural patterns in the vmPFC during eCFT may become more similar to the original memory over time due to the reengagement of this same scene construction process, irrespective of how the memory is being manipulated.

While both these possibilities may be true, it is also worth noting that researchers have long acknowledged a multifaceted role for the ventral PFC in a myriad of processes relevant to the current study, such as facilitating memory specificity, emotional processing, and self-awareness^36^. Research on neurologic and psychiatric conditions offers particularly relevant findings of episodic memory distortions resulting from PFC dysfunction. Patients with lesions to the vmPFC and OFC, for instance, find it difficult to dissociate between real and imagined events^37^, typically present with a lack of self-insight, self-reflection, and regret^38,39^, and report less specific details on episodic tasks^40^. Relatedly, clinical depression—which is often associated with aberrant activity in the vmPFC^41^—is also marked by difficulty recalling specific event details for autobiographical memories^42^. In addition to these roles in memory processes, vmPFC activity is also frequently implicated in representing subjective value and guiding the evaluation of outcomes between real and counterfactual events^43^. In the present study, then, increasing Recall-CFT NPS in the ventral PFC may also track comparisons between the emotionality of the real outcome with the counterfactual alternative (whether it’s a better or worse outcome). This interpretation is further supported by evidence linking multivariate representations in the vmPFC to competition among multiple value expectations^44^.

To adjudicate between these various possibilities, future research would benefit from examining whether the strength of change in neural patterns scales with the degree to which a memory is counterfactually modified. We were unable to test this possibility, given that we allowed participants to freely counterfactually modify their memories as they wished (we only specified the direction of counterfactual change), and did not collect feedback from participants on exactly what was being mentally altered. We hypothesize that greater change to the episodic features of the original memory would result in more dissimilar patterns in parietal regions such as the AG and SMG. Less change to the episodic features of the memory may result in more similar neural patterns in ventral PFC (as the original memory is being maintained), whereas the lack of such an effect may instead indicate a more domain-general process associated with NPS in this region (such as scene construction). Alternatively, an increase in recall-CFT NPS within the ventral PFC may be stronger while individuals specifically focus on affective comparisons between the simulated counterfactual and the original memory, and/or generate counterfactuals that specifically manipulate self-referential (internal) aspects of what occurred as opposed to circumstantial (external) details^23^.

Examining these possibilities will further clarify the effects reported here. Ultimately, though, our NPS findings suggest that the posterior parietal cortex and ventral PFC—two regions frequently implicated in eCFT—differentially guide the generation of counterfactual episodes despite both generally exhibiting increased neural activation at a univariate level of analysis. Multivariate patterns in the AG and SMG become more dissimilar from the original autobiographical memory as novel episodic details are integrated to construct a counterfactual outcome, whereas neural patterns in the vmPFC and lateral OFC become more similar to the original memory presumably to retain episodic aspects of the original event, reengage similar processes that support episodic simulation, or reflect on affective and self-referential aspects of the simulated counterfactual.

### Recall-CFT and CFT–CFT similarity are moderated by trait anxiety

Our findings of decreased Recall-CFT NPS in the posterior parietal cortex and increased similarity in the ventral PFC were not sensitive to individual differences in trait anxiety, or the type of eCFT. However, we did identify a distributed set of regions that differentially shifted in NPS depending on the interaction of these two factors, including the inferior frontal gyrus, superior frontal gyrus, posterior cingulate cortex, and cerebellum. For all these regions, individuals with higher trait anxiety exhibited a greater increase in Recall-CFT NPS for upward eCFT than downward eCFT, and vice versa for those with lower trait anxiety. In other words, these regions represent recapitulation of the original neural signature for an autobiographical memory, which seems to be more resistant to change for high-anxiety upward eCFT and low-anxiety downward eCFT. We interpret these effects as suggesting that individuals with high anxiety tend to simulate positive alternatives (upward eCFT) with less differentiation from the original autobiographical memory, whereas those with low anxiety tend to simulate negative alternatives (downward eCFT) with less differentiation from the original autobiographical memory. These findings converge with behavioral evidence that has shown anxious individuals to specifically generate less detailed counterfactuals of better outcomes and also believe better counterfactual outcomes to be less likely to occur^14^. In a related vein, a meta-analysis on the relationship between anxiety and episodic future thinking found that increased anxiety is consistently associated with less detailed simulations when cued by positively-valenced cues, but more detailed simulations when cued by negatively-valenced cues^45^. These anxiety-related effects diverge from what is seen in the general population, where studies have shown that people are, on average, more prone to simulating positive future events^46^ and generating upward counterfactuals of past experiences^47^. In fact, higher dispositional optimism positively predicts more upward eCFT^48^. This bias is thought to reflect the utility of imagining positive alternatives to past events in order to facilitate future behavior that helps us reach desirable goals^47^.

In summary, our results indicate that individuals with low anxiety tend to simulate better counterfactuals that are more distinct in neural patterns from the original memory than worse counterfactuals, reflecting a bias for generating more distinct varieties of better alternatives that has been well-documented in the literature. By comparison, individuals with high anxiety tend to simulate worse counterfactuals that are more distinct in neural patterns from the original memory than better counterfactuals, reflecting a bias for generating more distinct varieties of worse alternatives. That these effects were specifically observed in the inferior frontal gyrus, superior frontal gyrus, posterior cingulate cortex, and cerebellum illustrates that other regions within the broader DMN are also shifting in neural patterns during eCFT^49^, but not consistently across subjects and conditions.

To further test the relationship between anxiety and eCFT-related NPS, we also examined the similarity of neural patterns for counterfactuals belonging to the same condition (upward or downward). In this analysis, instead of comparing the neural patterns between an autobiographical memory and its subsequent counterfactual, we compared counterfactuals to one another. Again, we found evidence indicating that increased anxiety is associated with more generalized representations of better alternatives than worse alternatives, primarily within DMN regions. That is, as anxiety increased across participants, better counterfactuals became more similar to one another in their neural signature than worse counterfactuals. The only main effect we observed was in the right caudate nucleus, which exhibited higher NPS among both upward and downward eCFT conditions with increasing anxiety. Previous studies have suggested a general role for the dorsal striatum in subserving a number of mental simulation processes, including eCFT, future thinking, and visual perspective shifts^21,50,51^. Our present findings may therefore indicate that while anxious individuals are especially prone to simulating generalized better counterfactual outcomes, they also exhibit a tendency for all mental simulations to be slightly less differentiated in content (specifically within the caudate). This interpretation aligns with prior behavioral work showing that anxious individuals generally tend to simulate hypothetical events with less detail^52^.

### Limitations and future directions

Here we applied a novel multivariate assessment of neural patterns associated with autobiographical recall and eCFT, and in doing so identified regions that shift in the similarity of their neural patterns as participants generated counterfactual outcomes, as well as other DMN regions that differentially shift in NPS depending on the direction of the counterfactual and an individual’s trait anxiety. We interpreted these NPS effects as reflective of the counterfactual process whereby the outcome of an episodic event is modified. However, given the novelty of this approach, more work is needed to verify these claims. For instance, we did not collect ratings of subjective detail/vividness while participants engaged in eCFT, and future work will benefit from correlating such ratings with shifting neural patterns to more precisely discern whether changes in NPS indeed track the discriminability and distinctiveness of counterfactual simulations. Similarly, future studies may instruct participants to verbalize the content of their autobiographical memories and counterfactual simulations in order to verify the counterfactual modifications to memory and to better understand how these mental processes unfold over time. However, one study directly compared the brain activation patterns for silent and verbalized recall of autobiographical memories, finding some differences in key regions of autobiographical memory retrieval such as the AG, PCC, and hippocampus^53^, and therefore the implications of using verbalized recall need to be carefully considered.

Moreover, we show here that trait anxiety significantly moderated shifting neural patterns in many DMN regions, although we only obtained a self-reported measure of anxiety and did not specifically assess for any anxiety-related disorders. It is important to note, though, that nearly half of our participants were classified as highly anxious (STAI-Y2 scores ≥ 40), providing an appropriate distribution of scores to test the moderating role of anxiety^54^. Additionally, all analyses employed linear mixed effects modeling, which allows for trial-specific analyses and provides increased power compared to regression analyses that do not model random effects. Nevertheless, our final sample size was limited in part because we aimed to recruit participants from a wide spectrum of trait anxiety scores. Given recent discussions on the challenges in finding associations between individual differences and fMRI activity^55^ and the increasing amount of behavioral work on this topic, future work should continue to examine the role of anxiety in shaping multivariate neural patterns associated with eCFT in larger samples and with clinical groups.

Finally, it remains unclear the lasting consequences of eCFT on subsequent retrievals of autobiographical memory. That is, here we focused on neural patterns associated with generating a counterfactual outcome immediately after autobiographical recall, although we do not know whether such patterns actually integrated with the original memory to alter its behavioral or neural profile at subsequent retrieval sessions. Such an assessment likely requires multiple experiences with the same counterfactual simulation to significantly alter the original memory. Alternatively, the effects of eCFT might be amplified if participants are separated into groups who repeatedly generate only better or worse counterfactual outcomes^9^. Pinpointing how eCFT leads to long-term changes in the phenomenological experience and neural profile of a negative memory has profound clinical applications^1^, although very little research has been done in this area^7^ and thus remains an exciting area for future research to address.

## Methods

### Participants

Healthy adults were recruited through online and physical flyers. Participants were eligible if they were below 30 years of age, were right-handed, had no history of a psychiatric or neurological condition, and spoke English as their first language. We prescreened participants with the State-Trait Anxiety Inventory Form Y-2 (STAI-Y2)^56^ to determine their trait anxiety level. We aimed to recruit at least 15 individuals with high levels of anxiety (STAI-Y2 score ≥ 40) and at least 15 with low levels of anxiety, based on our previous work looking at group differences with counterfactual thinking^57^. Fifty-four healthy adults were initially recruited. Of these, fifteen participants were excluded from the final analysis due to participant drop out (*n* = 5), scanner failure (*n* = 8), recent participation in a similar eCFT study (*n* = 1), or lack of adherence to the task (*n* = 1), leaving a final sample of 39 participants (28 female; age = 22.67 ± 3.20; 20 Caucasian/White, 9 Asian/Pacific Islander, 3 Black/African American, 6 Latino/Hispanic, 1 Native American; STAI-Y2 = 40.21 ± 9.66; 17 “highly anxious”). Our sample size is comparable to previous studies examining the multivariate activity patterns related to autobiographical memory or counterfactual thinking^9,21,23^. All participants provided written informed consent before taking part in the study and received monetary compensation for their time. The study protocol was approved by the Duke University Health System Institutional Review Board, and all methods were performed in accordance with the relevant guidelines and regulations.

### Procedure

The experiment consisted of three sessions. In Session 1, participants entered the lab and recalled 45 autobiographical memories of specific regretful decisions they had made in the past 5 years. Participants were given a previously normed list of 50 common decisions such as “Drinking at a party” for inspiration^7,57^. For each memory, participants wrote a brief description of the event, created a short title for the memory (to be used as a cue during fMRI), and rated multiple phenomenological characteristics of their memory: valence (1 = negative to 7 = positive), arousal (1 = calm to 7 = excited), level of detail with which the memory was remembered (1 = vague to 7-clear), and how frequently they thought of the memory (1 = once a year or less to 7 = daily).

Session 2 occurred in the MRI scanner. In Session 2, which occurred one week after Session 1, participants were cued with memory titles to recall their own autobiographical memories (see Fig. 1a). For the first 12 s, participants recalled the original event as it occurred. Each autobiographical memory was assigned to one of three conditions: for 15 memories, participants were subsequently asked to simulate an *upward (better)* counterfactual event for another 12 s; for 15 memories, participants were subsequently asked to simulate a *downward (worse)* counterfactual event for another 12 s; for the remaining 15 memories, participants were not asked to generate any counterfactual thoughts during Session 2, and the trial ended after the 12-s autobiographical memory recall period. For trials in both upward and downward eCFT conditions, autobiographical memory recollection and eCFT manipulation were separated by a jittered fixation cross screen that lasted for 1, 2, or 3 s. During both autobiographical memory recollection and eCFT simulation, participants were instructed to press a button to indicate once they had recalled the memory/generated a counterfactual outcome. Trials were separated by a fixation cross screen for 1.5 s and an active numerical task on which participants saw two to four numbers between 0 and 99 for 1.5 s each and judged whether the numbers were odd or even. A final session occurred 1 day after, during which participants were cued with memory titles to recall their own autobiographical memories and rated the phenomenological characteristics. The results of this session, however, are outside of the aims of this study and are thus reported separately.

### fMRI data acquisition and preprocessing

All fMRI scanning sessions were completed at the Brain Imaging and Analysis Center at Duke University using a 3 T GE MR750 Scanner. Foam padding was placed inside the head coil to minimize head movement by participants. Each scanning session began with a localizer and a high-resolution T1-weighted structural scan (162 1-mm isotropic slices, TR = 8.16 ms, TE = 3.18 ms), followed by three functional scans using a whole brain, gradient-echo, spiral-in sequence (TR = 2 s, TE = 30 ms, FOV = 240 mm, matrix size = 64 × 64, flip angle = 80°). Slices were acquired in an interleaved fashion (36 3.8 mm axial isotropic slices, slice gap of 0.076 mm). Experimental task instructions were presented using Psychtoolbox software. Each functional task began with eight seconds (4 TRs) of fixation to allow MR scanner stabilization and those timepoints were discarded prior to preprocessing. Participant responses were made on two 4-button response boxes held in each hand. All fMRI analyses were performed on data collected during Session 2.

#### Anatomical data preprocessing

All preprocessing steps were performed using fMRIPrep 21.0.1^58^ (RRID:SCR_016216). T1-weighted anatomical images were corrected for intensity non-uniformity with N4BiasFieldCorrection^59^, distributed with ANTs 2.3.3^60^ (RRID:SCR_004757). The T1-weighted reference was then skull-stripped with a Nipype implementation of the antsBrainExtraction.sh workflow from ANTs, using OASIS30ANTs as target template. Brain tissue segmentation of cerebrospinal fluid, white-matter, and gray-matter was performed on the brain-extracted T1-weighted images using fast (FSL 6.0.5.1:57b01774, RRID:SCR_002823)^61^. A T1-weighted reference map was computed after registration of 2 T1-weighted images using mri_robust_template from FreeSurfer 6.0.1^62^. Volume-based spatial normalization was performed through nonlinear registration with antsRegistration, to FSL’s MNI ICBM 152 non-linear 6th Generation Asymmetric Average Brain Stereotaxic Registration Model (MNI152NLin6Asym) [RRID:SCR_002823]^63^.

#### Functional data preprocessing

For each of the three functional imaging runs per subject, the following preprocessing steps were performed. First, a reference volume and its skull-stripped version were generated using fMRIPrep. Head-motion parameters with respect to the blood-oxygenation-level-dependent (BOLD) signal reference (transformation matrices, and six corresponding rotation and translation parameters) were estimated before any spatiotemporal filtering using mcflirt (FSL 6.0.5.1:57b01774)^64^. Functional imaging runs were slice-time corrected to 0.972 s (0.5 of slice acquisition range 0–1.94 s) using 3dTshift from AFNI (RRID:SCR_005927)^65^. The BOLD time-series were resampled onto each participant’s native space by applying the transforms to correct for head-motion. The BOLD reference was then co-registered to the T1-weighted reference using mri_coreg (FreeSurfer) followed by flirt (FSL 6.0.5.1:57b01774)^66^ with the boundary-based registration^67^ cost-function. Co-registration was configured with six degrees of freedom. Several confounding time-series were calculated based on the preprocessed BOLD data: framewise displacement, DVARS, and three region-wise global signals. Framewise displacement was computed using two formulations following Power (absolute sum of relative motions)^68^ and Jenkinson (relative root mean square displacement between affines)^64^. Framewise displacement and DVARS are calculated for each functional run, both using their implementations in Nipype. The three global signals are extracted within the cerebrospinal fluid, the white matter, and the whole-brain masks. The confound time series derived from head motion estimates and global signals were expanded with the inclusion of temporal derivatives and quadratic terms for each^69^. Frames that exceeded a threshold of 0.5-mm framewise displacement or 1.5 standardized DVARS were annotated as motion outliers. The BOLD time-series data were resampled into standard space, generating a preprocessed BOLD run in MNI152NLin6Asym space. First, a reference volume and its skull-stripped version were generated using a custom methodology of fMRIPrep. All resamplings can be performed with a single interpolation step by composing all the pertinent transformations (i.e., head-motion transform matrices, susceptibility distortion correction when available, and co-registrations to anatomical and output spaces). Gridded (volumetric) resamplings were performed using antsApplyTransforms from ANTs, configured with Lanczos interpolation to minimize the smoothing effects of other kernels^70^. Non-gridded (surface) resamplings were performed using mri_vol2surf from FreeSurfer. The denoised outputs from fMRIPrep were skull-stripped using FSL’s brain extraction tool and high-pass filtered at 100 s.

### Univariate analysis

Univariate analyses were performed using FSL’s FEAT^71^. First-level general linear models (GLMs) were constructed for each subject and run using task regressors and corresponding temporal derivatives based on timing onsets and durations for the initial recall of each memory, generating worse counterfactuals, generating better counterfactuals, completing the even/odd fixation task, and button presses (to account for motor-related activity). We also included confound regressor time series for cerebrospinal fluid, white matter, DVARS, framewise displacement, and six translation and rotation motion parameters, as well as any censored timepoints (framewise displacement > 0.5 or standardized DVARS > 1.5). Regressors were convolved with a double-gamma hemodynamic response function. Given that the univariate analyses were performed to isolate a set of regions that exhibit increasing or decreasing activation during eCFT (to be further examined with multivariate analyses), we used the same preprocessed input data (without smoothing) in both univariate and multivariate analyses to maintain consistency.

Functional runs were combined within-subject at second-level analyses using fixed effects. The resulting maps were then combined at the group level using FMRIB’s local analysis of mixed effects (FLAME 1 + 2 with outlier deweighting). We computed a single-group average (one-sample *t*-test) to evaluate the average response for each contrast originally defined at the first-level. Group results were assessed at a cluster-forming threshold of z = 2.3 and a cluster significance threshold of α = 0.05.

### Multivariate neural pattern similarity analyses

We next conducted a novel investigation of the multi-voxel activity patterns elicited by recalling autobiographical events and simulating different counterfactual episodes. For these analyses, we parcellated the whole brain into non-overlapping regions based on the Automated Anatomical Labelling atlas 3^72^. Fifty-six regions of interest (ROIs) were selected based on two criteria: 1) containing at least 100 voxels—our high-pass threshold for examining a region’s multi-voxel activity patterns, and 2) having at least 25% of their voxels identified as significantly different across conditions in the previous omnibus test on univariate activation (see Supplementary Table S1).

For trial-level neural pattern similarity analyses we constructed similar GLMs as we did for the univariate analyses, but modeled each trial of interest as a separate regressor in line with the Least Squares Separate approach^73^. Specifically, an instance of either autobiographical memory recall or directed eCFT was modeled using a finite impulse response (FIR) function^74,75^ with three 4-s windows (i.e., two TRs; see Supplementary Fig. S1b). The FIR modeling approach was chosen over the canonical double-Gamma hemodynamic response function to better characterize the temporal dynamics of recollections and mental simulations over an extended period of time (i.e., 12 s), as in other studies of autobiographical memory with extended recall phases^76,77^. Therefore, for each instance of autobiographical memory recall or eCFT we obtained three parameters, corresponding to 0–4 s, 4–8 s, and 8–12 s on that trial. Five regressors of no interest were also included, specifying the following events in the same BOLD run: (i) all other instances of autobiographical memory recall, (ii) all other instances of upward eCFT simulation, (iii) all other instances of downward eCFT simulation, (iv) odd/even numerical judgment tasks, and (v) button presses on all tasks (duration set to 0.1 s). These regressors were convolved with the double-Gamma hemodynamic response function and we also included their temporal derivatives to allow for variations in the exact timing of peak response. Additionally, covariates of no interest included BOLD signals from CSF and WM, and motion-related time series including six motion parameters (translational and rotational), DVARS, framewise displacement, and motion outlier indicators estimated by fMRIPrep for each TR. Parameter estimates from models whose focal event contained three or more (out of six) motion outlier TRs were excluded from analyses.

Three multi-voxel patterns of the *t*-statistics of parameter estimates were gathered from each ROI for each trial in the directed eCFT conditions. The three patterns corresponded to autobiographical memory recall time-window (4–8 s), eCFT time-window (0–4 s) and eCFT time-window (4–8 s). The similarity between each pair of patterns, *neural pattern similarity* (NPS), was quantified as their Fisher-transformed Pearson correlation coefficient. We computed two summary measures of NPS—one based on the similarity between recall and eCFT of the same autobiographical event and the other based on the similarity between different instances of eCFT.

#### Recall-CFT similarity shift index

First, we computed a “Recall-CFT similarity shift” index that quantifies how much an ROI’s activity pattern of counterfactual episode diverged from that of the original autobiographical event as participants elaborated on their simulations. For each instance of autobiographical memory recall, we focused on its activity pattern during 4–8 s, which is when participants were elaborating on the details from the event; for each instance of eCFT simulation, we focused on the activity patterns during 0–4 s and during 4–8 s during which the counterfactual outcome was being generated and elaborated upon. Then, for each trial we computed a Recall-CFT similarity shift index (see Fig. 1b) according to the following formula:

$${\text{[Recall-CFT similarity shift]}}_{x}=z\left(r\left({\text{Recall}}_{x, \, \left[\text{4-8 s}\right]}, {\text{CFT}}_{x, \, \left[\text{4-8 s}\right]}\right)\right)-z\left(r\left({\text{Recall}}_{x, \, \left[\text{4-8 s}\right]}, {\text{CFT}}_{x, \, \left[\text{0-4 s}\right]}\right)\right).$$

In addition, we ran a linear regression model to estimate the effects of Recall-CFT task interval (1 s, 2 s, or 3 s) and the previous trial condition (upward eCFT, downward eCFT, No eCFT, or no previous trial), which were removed from the raw Recall-CFT similarity shift index to account for autocorrelation and any lasting effects of the previous trial, respectively.

Of note, the selection of Recall and CFT windows in the calculation of Recall-CFT similarity shift index were motivated by both theoretical and practical reasons. For both Recall and CFT, participants were primarily constructing their autobiographical memories or counterfactuals during the first window (0–4 s), while elaborating on their thoughts in the following windows (4–8 s) and (8–12 s) (see Supplementary Fig. S1b). Due to the relatively short temporal separation between Recall and CFT tasks (a fixation screen jittered between 1 and 3 s), NPS values between Recall (8–12 s) and CFT (0–4 s) would be highly inflated due to the temporal autocorrelation of BOLD signals and were therefore disregarded. Thus, we only considered the neural activity pattern from the middle Recall window (4–8 s) as the representation of autobiographical memories. Meanwhile, we did not use the similarity between Recall (4–8 s) and CFT (8–12 s) because this pair of windows were separated by almost twice as long as the separation between Recall (4–8 s) and CFT (0–4 s), which would render interpretation difficult. Nevertheless, we provide the neural pattern similarity values between Recall (4–8 s) and all three CFT windows for a subset of brain regions (see Supplementary Fig. S3).

Separate linear mixed-effects models (LMEMs) were fitted to examine the Recall-CFT similarity shift index from each of our 56 ROIs. Specifically, our models included fixed effects of each trial’s eCFT condition (upward vs. downward), participant trait anxiety level, baseline arousal rating of each autobiographical event, and all associated interaction terms. All continuous predictor variables were mean-centered and standardized. Participant-level random intercepts were also estimated. Of note, inter-task interval and the previous trial condition were again included as nuisance regressors such that results from our two-step regression approach can be interpreted the same way as results from a standard regression model with all fixed effects predicting the original dependent variable^78,79^. P-values were adjusted for false discovery rate^80^. Post hoc tests for significant interactions were examined using the ‘emmeans’ package^81^. This analysis aimed to identify ROIs whose activity patterns either demonstrated CFT-induced shift or actively maintained the original event.

#### CFT-CFT similarity index

Our second NPS summary measure, CFT-CFT similarity index, examined the specificity of each counterfactual simulation with respect to other simulations of the same type. For this analysis, we focused on NPS values based on 4–8 s of eCFT when participants were elaborating on their simulated content to be consistent with the previous analysis on Recall-CFT similarity shift. Separately for upward and downward eCFT conditions, we used the following formula to compute CFT-CFT similarity between one eCFT instance and all other instances:

$${\text{[CFT-CFT similarity]}}_{x}=\frac{1}{m}\sum_{j\ne x}z \left(r\left({\text{CF}}{\text{T}}_{x}, {\text{CF}}{\text{T}}_{j}\right)\right),$$

where $m$ is the total number of other eCFT instances in the same condition but from a different fMRI run (see Fig. 1b). NPS values based on activity patterns from the same run were excluded to circumvent inflated similarity estimates due to BOLD signal autocorrelation. A high CFT-CFT similarity index indicates that a given counterfactual simulation was similar to others of the same type, suggesting that the simulation had a generalized neural representation. On the contrary, a low CFT–CFT similarity suggests greater specificity of the counterfactual content.

Separate LMEMs were fitted to examine the CFT–CFT similarity index from each ROI. Specifically, our models included fixed effects of each trial’s eCFT condition (upward vs. downward), participant trait anxiety level, baseline arousal ratings of the memory, and all associated interaction terms. Random intercepts were estimated for each participant. P-values were adjusted for false discovery rate^80^. Post hoc tests for significant interactions were examined using the ‘emmeans’ package^81^.

### Supplementary Information

Supplementary Information.

## Data Availability

The datasets analyzed for the current study are available from the corresponding author upon request.

## References

[1] De Brigard F, Parikh N (2019). Episodic counterfactual thinking. Curr. Dir. Psychol. Sci..

[2] Branch JG (2023). Individual differences in the frequency of voluntary & involuntary episodic memories, future thoughts, and counterfactual thoughts. Psychol. Res..

[3] Byrne RMJ (2002). Mental models and counterfactual thoughts about what might have been. Trends Cogn. Sci..

[4] Mandel D (2003). Counterfactuals, emotions, and context. Cogn. Emot..

[5] Kahneman D, Miller DT (1986). Norm theory: Comparing reality to its alternatives. Psychol. Rev..

[6] Roese NJ (1997). Counterfactual thinking. Psychol. Bull..

[7] De Brigard F, Hanna E, St Jacques PL, Schacter DL (2019). How thinking about what could have been affects how we feel about what was. Cogn. Emot..

[8] Parikh N, De Brigard F, LaBar KS (2022). The efficacy of downward counterfactual thinking for regulating emotional memories in anxious individuals. Front. Psychol..

[9] Speer ME, Ibrahim S, Schiller D, Delgado MR (2021). Finding positive meaning in memories of negative events adaptively updates memory. Nat. Commun..

[10] Koster EHW, De Lissnyder E, Derakshan N, De Raedt R (2011). Understanding depressive rumination from a cognitive science perspective: The impaired disengagement hypothesis. Clin. Psychol. Rev..

[11] Nolen-Hoeksema S, Wisco BE, Lyubomirsky S (2008). Rethinking rumination. Perspect. Psychol. Sci..

[12] Tagini S (2021). Counterfactual thinking in psychiatric and neurological diseases: A scoping review. PLOS ONE.

[13] Ruiselová Z, Prokopcáková A, Kresánek J (2009). Counterfactual thinking as a coping strategy—Cognitive and emotional aspects. Stud. Psychol..

[14] Parikh N, LaBar KS, De Brigard F (2020). Phenomenology of counterfactual thinking is dampened in anxious individuals. Cogn. Emot..

[15] Schwabe L, Wolf OT (2009). New episodic learning interferes with the reconsolidation of autobiographical memories. PLOS ONE.

[16] Walker, M. P., Brakefield, T., Allan Hobson, J. & Stickgold, R. Dissociable stages of human memory consolidation and reconsolidation. *Nature* **425**, 616–620 (2003). 10.1038/nature01930 14534587

[17] De Brigard F, Addis DR, Ford JH, Schacter DL, Giovanello KS (2013). Remembering what could have happened: Neural correlates of episodic counterfactual thinking. Neuropsychologia.

[18] Van Hoeck N (2013). Counterfactual thinking: An fMRI study on changing the past for a better future. Soc. Cogn. Affect. Neurosci..

[19] Addis DR, Schacter DL (2008). Constructive episodic simulation: Temporal distance and detail of past and future events modulate hippocampal engagement. Hippocampus.

[20] Parikh N, Ruzic L, Stewart GW, Spreng RN, De Brigard F (2018). What if? Neural activity underlying semantic and episodic counterfactual thinking. NeuroImage.

[21] Faul, L., St. Jacques, P. L., DeRosa, J. T., Parikh, N. & De Brigard, F. Differential contribution of anterior and posterior midline regions during mental simulation of counterfactual and perspective shifts in autobiographical memories. *NeuroImage* **215**, 116843 (2020). 10.1016/j.neuroimage.2020.116843 32289455

[22] De Brigard, F., Nathan Spreng, R., Mitchell, J. P. & Schacter, D. L. Neural activity associated with self, other, and object-based counterfactual thinking. *NeuroImage* **109**, 12–26 (2015). 10.1016/j.neuroimage.2014.12.075 PMC4710471 25579447

[23] Khoudary A (2022). Neural differences between internal and external episodic counterfactual thoughts. Philos. Trans. R. Soc. B Biol. Sci..

[24] Kriegeskorte, N., Mur, M. & Bandettini, P. Representational similarity analysis—Connecting the branches of systems neuroscience. *Front. Syst. Neurosci.* **2**, 4 (2008). 10.3389/neuro.06.004.2008 PMC2605405 19104670

[25] Shimamura AP (2011). Episodic retrieval and the cortical binding of relational activity. Cogn. Affect. Behav. Neurosci..

[26] Ramanan S, Piguet O, Irish M (2018). Rethinking the role of the angular gyrus in remembering the past and imagining the future: The contextual integration model. Neuroscientist.

[27] McCormick C, Barry DN, Jafarian A, Barnes GR, Maguire EA (2020). vmPFC drives hippocampal processing during autobiographical memory recall regardless of remoteness. Cereb. Cortex.

[28] Lin W-J, Horner AJ, Burgess N (2016). Ventromedial prefrontal cortex, adding value to autobiographical memories. Sci. Rep..

[29] Kocovski NL, Endler NS, Rector NA, Flett GL (2005). Ruminative coping and post-event processing in social anxiety. Behav. Res. Ther..

[30] Monforton J, Vickers K, Antony MM (2012). “If only I didn’t embarrass myself in front of the class!”: Social anxiety and upward counterfactual thinking. J. Soc. Clin. Psychol..

[31] Gagne C, Dayan P, Bishop SJ (2018). When planning to survive goes wrong: Predicting the future and replaying the past in anxiety and PTSD. Curr. Opin. Behav. Sci..

[32] Thakral PP, Madore KP, Kalinowski SE, Schacter DL (2020). Modulation of hippocampal brain networks produces changes in episodic simulation and divergent thinking. Proc. Natl. Acad. Sci..

[33] St. Jacques, P. L., Szpunar, K. K. & Schacter, D. L. Shifting visual perspective during retrieval shapes autobiographical memories. *NeuroImage* **148**, 103–114 (2017). 10.1016/j.neuroimage.2016.12.028 PMC5344759 27989780

[34] Bertossi E, Aleo F, Braghittoni D, Ciaramelli E (2016). Stuck in the here and now: Construction of fictitious and future experiences following ventromedial prefrontal damage. Neuropsychologia.

[35] McCormick C, Ciaramelli E, De Luca F, Maguire EA (2018). Comparing and contrasting the cognitive effects of hippocampal and ventromedial prefrontal cortex damage: A review of human lesion studies. Neuroscience.

[36] Brand, M. & Markowitsch, H. J. Memory processes and the orbitofrontal cortex. In *The Orbitofrontal Cortex* (eds. Zald, D. & Rauch, S.). 10.1093/acprof:oso/9780198565741.003.0011 (Oxford University Press, 2006).

[37] Hebscher M, Gilboa A (2016). A boost of confidence: The role of the ventromedial prefrontal cortex in memory, decision-making, and schemas. Neuropsychologia.

[38] Barrash J, Tranel D, Anderson SW (2000). Acquired personality disturbances associated with bilateral damage to the ventromedial prefrontal region. Dev. Neuropsychol..

[39] Camille N (2004). The involvement of the orbitofrontal cortex in the experience of regret. Science.

[40] Bertossi E, Candela V, De Luca F, Ciaramelli E (2017). Episodic future thinking following vmPFC damage: Impaired event construction, maintenance, or narration?. Neuropsychology.

[41] Borsini A, Wallis ASJ, Zunszain P, Pariante CM, Kempton MJ (2020). Characterizing anhedonia: A systematic review of neuroimaging across the subtypes of reward processing deficits in depression. Cogn. Affect. Behav. Neurosci..

[42] Sumner JA, Griffith JW, Mineka S (2010). Overgeneral autobiographical memory as a predictor of the course of depression: A meta-analysis. Behav. Res. Ther..

[43] Coricelli G, Dolan RJ, Sirigu A (2007). Brain, emotion and decision making: The paradigmatic example of regret. Trends Cogn. Sci..

[44] Moneta N, Garvert MM, Heekeren HR, Schuck NW (2023). Task state representations in vmPFC mediate relevant and irrelevant value signals and their behavioral influence. Nat. Commun..

[45] Du JY, Hallford DJ, Busby Grant J (2022). Characteristics of episodic future thinking in anxiety: A systematic review and meta-analysis. Clin. Psychol. Rev..

[46] Salgado S, Berntsen D (2020). My future is brighter than yours: The positivity bias in episodic future thinking and future self-images. Psychol. Res..

[47] Epstude K, Roese NJ (2008). The functional theory of counterfactual thinking. Pers. Soc. Psychol. Rev..

[48] Gamlin J, Smallman R, Epstude K, Roese NJ (2020). Dispositional optimism weakly predicts upward, rather than downward, counterfactual thinking: A prospective correlational study using episodic recall. PLOS ONE.

[49] Buckner RL, DiNicola LM (2019). The brain’s default network: Updated anatomy, physiology and evolving insights. Nat. Rev. Neurosci..

[50] D’Argembeau A, Xue G, Lu Z-L, Van der Linden M, Bechara A (2008). Neural correlates of envisioning emotional events in the near and far future. NeuroImage.

[51] Szpunar, K. K., St. Jacques, P. L., Robbins, C. A., Wig, G. S. & Schacter, D. L. Repetition-related reductions in neural activity reveal component processes of mental simulation. *Soc. Cogn. Affect. Neurosci.* **9**, 712–722 (2014). 10.1093/scan/nst035 PMC4014108 23482621

[52] Wu JQ, Szpunar KK, Godovich SA, Schacter DL, Hofmann SG (2015). Episodic future thinking in generalized anxiety disorder. J. Anxiety Disord..

[53] Ferris CS, Inman CS, Hamann S (2024). FMRI correlates of autobiographical memory: Comparing silent retrieval with narrated retrieval. Neuropsychologia.

[54] Charpentier CJ (2021). How representative are neuroimaging samples? Large-scale evidence for trait anxiety differences between fMRI and behaviour-only research participants. Soc. Cogn. Affect. Neurosci..

[55] Dubois J, Adolphs R (2016). Building a science of individual differences from fMRI. Trends Cogn. Sci..

[56] Spielberger CD, Gorsuch RL, Lushene R, Vagg PR, Jacobs GA (1983). Manual for the State-Trait Anxiety Inventory.

[57] De Brigard F, Rodriguez DC, Montañés P (2017). Exploring the experience of episodic past, future, and counterfactual thinking in younger and older adults: A study of a Colombian sample. Conscious. Cogn. Int. J..

[58] Esteban O (2019). fMRIPrep: A robust preprocessing pipeline for functional MRI. Nat. Methods.

[59] Tustison NJ (2010). N4ITK: Improved N3 bias correction. IEEE Trans. Med. Imaging.

[60] Avants BB, Epstein CL, Grossman M, Gee JC (2008). Symmetric diffeomorphic image registration with cross-correlation: Evaluating automated labeling of elderly and neurodegenerative brain. Med. Image Anal..

[61] Zhang Y, Brady M, Smith S (2001). Segmentation of brain MR images through a hidden Markov random field model and the expectation-maximization algorithm. IEEE Trans. Med. Imaging.

[62] Reuter M, Rosas HD, Fischl B (2010). Highly accurate inverse consistent registration: A robust approach. NeuroImage.

[63] Evans AC, Janke AL, Collins DL, Baillet S (2012). Brain templates and atlases. NeuroImage.

[64] Jenkinson M, Bannister P, Brady M, Smith S (2002). Improved optimization for the robust and accurate linear registration and motion correction of brain images. NeuroImage.

[65] Cox RW, Hyde JS (1997). Software tools for analysis and visualization of fMRI data. NMR Biomed..

[66] Jenkinson M, Smith S (2001). A global optimisation method for robust affine registration of brain images. Med. Image Anal..

[67] Greve DN, Fischl B (2009). Accurate and robust brain image alignment using boundary-based registration. NeuroImage.

[68] Power JD (2014). Methods to detect, characterize, and remove motion artifact in resting state fMRI. NeuroImage.

[69] Satterthwaite TD (2013). An improved framework for confound regression and filtering for control of motion artifact in the preprocessing of resting-state functional connectivity data. NeuroImage.

[70] Lanczos, C. A precision approximation of the gamma function. *J. Soc. Ind. Appl. Math. Ser. B Numer. Anal.* **1**, 86–96 (1964).

[71] Woolrich MW, Ripley BD, Brady M, Smith SM (2001). Temporal autocorrelation in univariate linear modeling of FMRI data. NeuroImage.

[72] Rolls ET, Huang C-C, Lin C-P, Feng J, Joliot M (2020). Automated anatomical labelling atlas 3. NeuroImage.

[73] Mumford JA, Turner BO, Ashby FG, Poldrack RA (2012). Deconvolving BOLD activation in event-related designs for multivoxel pattern classification analyses. NeuroImage.

[74] Bai, B., Kantor, P. & Shokoufandeh, A. Effectiveness of the finite impulse response model in content-based fMRI image retrieval. In *Medical Image Computing and Computer-Assisted Intervention—MICCAI 2007* (eds. Ayache, N., Ourselin, S. & Maeder, A.). 742–750 10.1007/978-3-540-75759-7_90 (Springer, 2007). 10.1007/978-3-540-75759-7_90 18044635

[75] Goutte C, Nielsen FA, Hansen KH (2000). Modeling the hemodynamic response in fMRI using smooth FIR filters. IEEE Trans. Med. Imaging.

[76] Daselaar SM (2008). The spatiotemporal dynamics of autobiographical memory: Neural correlates of recall, emotional intensity, and reliving. Cereb. Cortex.

[77] Hall SA, Brodar KE, LaBar KS, Berntsen D, Rubin DC (2018). Neural responses to emotional involuntary memories in posttraumatic stress disorder: Differences in timing and activity. NeuroImage Clin..

[78] Chen W, Hribar P, Melessa S (2018). Incorrect inferences when using residuals as dependent variables. J. Acc. Res..

[79] Chen W, Hribar P, Melessa SJ (2022). On the use of residuals as dependent variables. J. Financ. Rep..

[80] Benjamini Y, Hochberg Y (1995). Controlling the false discovery rate: A practical and powerful approach to multiple testing. J. R. Stat. Soc. Ser. B Methodol..

[81] Lenth, R. *emmeans: Estimated Marginal Means, aka Least-Squares Means* (2020).

