# Supplementary material for: Counterfactual thinking induces different neural patterns of memory modification in anxious individuals
Source: Sci Rep. 2024 May 9;14:10630. doi: 10.1038/s41598-024-61545-x (PMC11082200; doi:10.1038/s41598-024-61545-x)
Supplement: Supplementary file 1 — Supplementary Information. [file 41598_2024_61545_MOESM1_ESM.docx]

## Counterfactual thinking induces different neural patterns of memory modification in anxious individuals

Shenyang Huang^1,2,+^, Leonard Faul^3,+^, Natasha Parikh^4^, Kevin S. LaBar^1,2^, Felipe De Brigard^1,2,5,^*

^1^Department of Psychology and Neuroscience, Duke University, Durham, NC 27708, USA;

^2^Center for Cognitive Neuroscience, Duke University, Durham, NC 27708, USA;

^3^Department of Psychology and Neuroscience, Boston College, Chestnut Hill, MA 02467, USA;

^4^Department of Psychology and Neuroscience, University of North Carolina at Chapel Hill, Chapel Hill, NC 27599, USA;

^5^Department of Philosophy, Duke University, Durham, NC 27708, USA.

*[felipe.debrigard@duke.edu](mailto:felipe.debrigard@duke.edu)

^+^these authors contributed equally to this work

## Supplementary information

### Figure S1. Phenomenological ratings of memories and recall/CFT response time

| 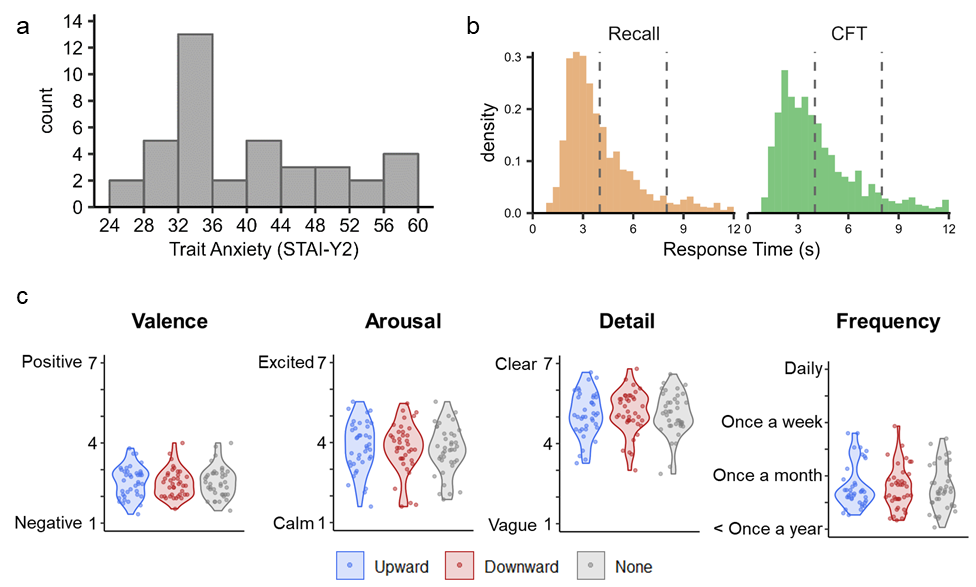 |
| --- |
| **Figure S1. (a)** *Trait anxiety scores*. Histogram of trait anxiety scores measured by the State-Trait Anxiety Inventory Form Y-2 (STAI-Y2). **(b)** *Response time*. For both autobiographical memory recall and counterfactual outcome simulation, participants were instructed to press a button to indicate when they had the memory or counterfactual thought in mind. In the majority of cases, within the first 4 seconds of recall/simulation participants had already recalled the event based on the verbal cue or generated a counterfactual outcome, therefore elaborating on the recalled or simulated contents for the rest of the allotted time. Thus, our neural pattern similarity (NPS) analyses focused on the middle, content-rich 4-second window (4-8 s). **(c)** *Phenomenological ratings of memories*. Violin plots of individual participant’s average ratings at Session 1, separately for memories randomly assigned to different episodic counterfactual thinking conditions at Session 2. |

### Figure S2. Whole-brain univariate analyses


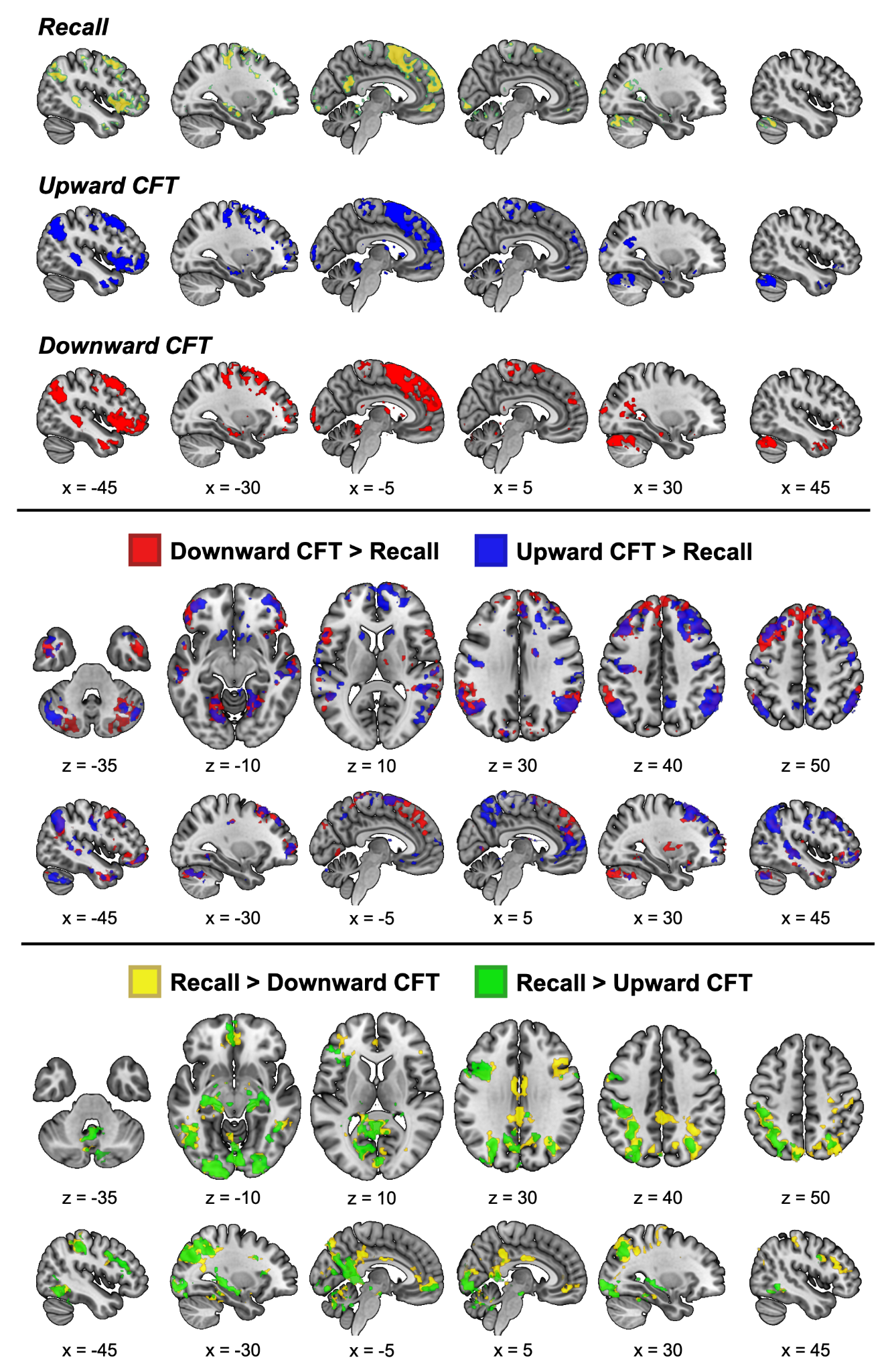


**Figure S2**. **Whole-brain univariate analyses.** Slices on the top row show the main effects of Recall, Upward CFT, and Downward CFT, whereas the middle and bottom rows show comparisons between conditions. All effects represent group results after mixed effects analysis (z ​> ​2.3, cluster-corrected P ​< 0​.05).

### Figure S3. Recall-CFT similarity

| 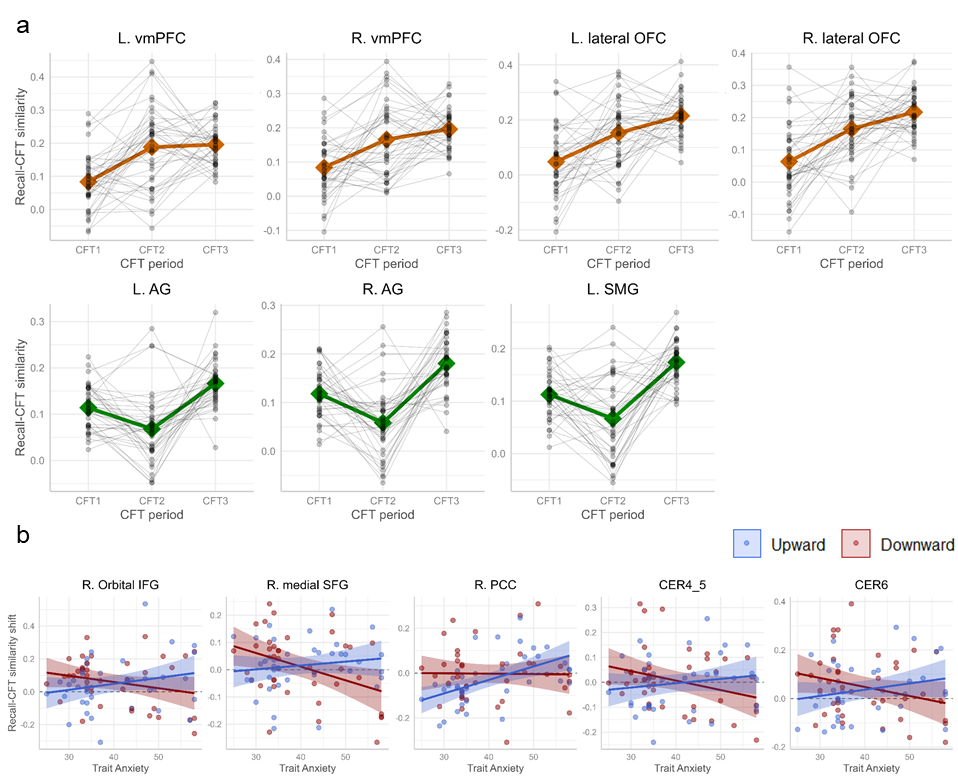 |
| --- |
| **Figure S3. Recall-CFT similarity (a)**. *Participant-level Recall-CFT similarity values for brain regions in* ***Figure 3a***. Gray circles indicate individual participants and colored diamonds indicate group means. Data points are linked across three CFT windows [0-4 s], [4-8 s], [8-12 s]. **(b)** *Participant-level Recall-CFT similarity shift index for brain regions in* ***Figure 3b***. Circles indicate individual participants and are colored by CFT conditions. Abbreviations: *vmPFC*, ventromedial prefrontal cortex; *OFC*, orbital frontal cortex; *SMG*, supramarginal gyrus; *AG*, angular gyrus; *IFG*, inferior frontal gyrus; *SFG*, superior frontal gyrus; *PCC*, posterior cingulate cortex; *CER4_5*, Lobule IV, V of cerebellar hemisphere; *CER6*, Lobule VI of cerebellar hemisphere. |

### Figure S4. CFT-CFT similarity

| 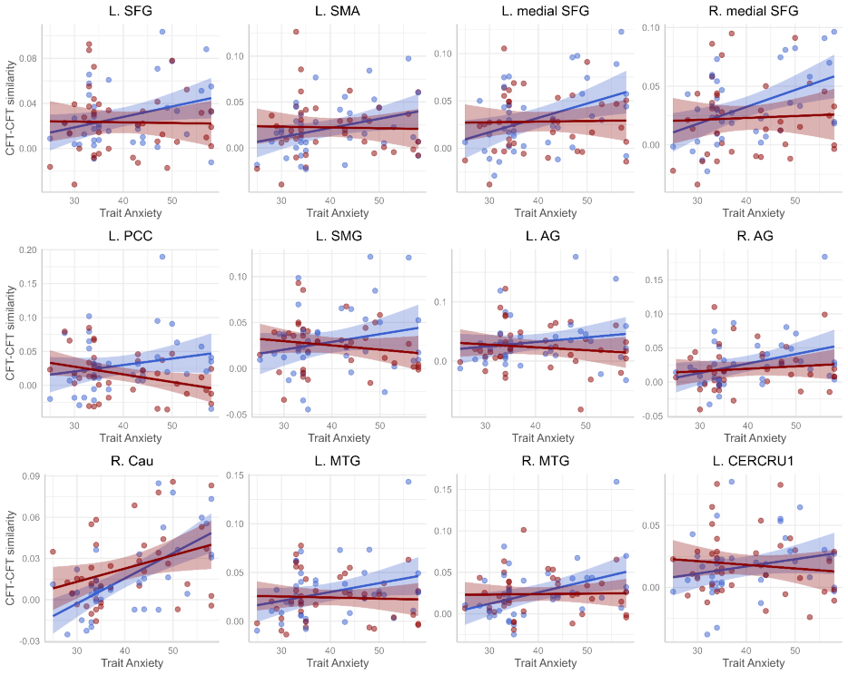 |
| --- |
| **Figure S4. CFT-CFT similarity**. Participant-level CFT-CFT similarity values for brain regions in **Figure 4a** are indicated by circles and colored by CFT conditions. Abbreviations: *SFG*, superior frontal gyrus; *SMA*, supplementary motor area; *PCC*, posterior cingulate cortex; *SMG*, supramarginal gyrus; *AG*, angular gyrus; *Cau*, caudate nucleus; *MTG*, Middle temporal gyrus; *CERCRU1*, Crus I of cerebellar hemisphere. |

### Table S1. Univariate analysis results. Local maxima (italicized) are reported for clusters greater than 5,000 voxels in size.

| **Region** | **Hemi** | **x** | **y** | **z** | **Z** | **Cluster Size** |
| --- | --- | --- | --- | --- | --- | --- |
| ***Downward CFT > Recall*** | | | | | | |
| MFG | L | -38 | 24 | 48 | 7.07 | 5238 |
| *MFG* | L | -34 | 22 | 44 | 6.02 |  |
| *MFG* | L | -40 | 18 | 50 | 6.00 |  |
| *SFG* | L | -10 | 16 | 66 | 5.51 |  |
| *SFG* | L | -16 | 26 | 60 | 5.45 |  |
| *SFG* | L | -16 | 32 | 56 | 5.32 |  |
| AG/LOC | L | -52 | -64 | 30 | 6.13 | 1521 |
| ITG | R | 50 | -2 | -38 | 6.24 | 1349 |
| ITG | L | -46 | -2 | -36 | 6.02 | 1118 |
| AG/LOC | R | 58 | -58 | 38 | 5.45 | 1032 |
| Crus I | R | 48 | -64 | -30 | 5.84 | 876 |
| Crus I | L | -28 | -80 | -28 | 5.89 | 608 |
| OFC | R | 48 | 30 | -10 | 4.99 | 595 |
| Frontal Pole | L | -44 | 50 | -12 | 5.92 | 555 |
| Lingual Gyrus | L | -18 | -56 | -8 | 5.69 | 472 |
| Lingual Gyrus | R | 16 | -60 | -6 | 4.94 | 433 |
| IFG | L | -54 | 20 | 6 | 5.12 | 374 |
| LOC | L | -18 | -88 | 26 | 5.36 | 310 |
| Caudate | R | 12 | 4 | 20 | 5.27 | 209 |
| Postcentral Gyrus | L | -30 | -20 | 38 | 4.08 | 195 |
| IFG | R | 56 | 20 | 10 | 4.10 | 138 |
| Postcentral Gyrus | L | -6 | -46 | 70 | 3.79 | 138 |
| LOC | R | 14 | -86 | 36 | 4.39 | 137 |
| Putamen | R | 34 | -10 | 2 | 3.83 | 136 |
| Precentral Gyrus | R | 38 | -12 | 40 | 4.15 | 131 |
| ***Upward CFT > Recall*** | | | | | | |
| MFG | L | -38 | 24 | 48 | 5.95 | 6540 |
| *MFG* | R | 30 | 18 | 58 | 5.88 |  |
| *MFG* | L | -38 | 24 | 44 | 5.87 |  |
| *MFG* | R | 40 | 24 | 46 | 5.87 |  |
| *Frontal Pole* | R | 8 | 64 | 2 | 5.74 |  |
| *Frontal Pole* | R | 18 | 36 | 32 | 5.65 |  |
| AG/LOC | L | -50 | -62 | 44 | 6.27 | 2423 |
| AG/LOC | R | 54 | -60 | 40 | 6.45 | 1801 |
| Postcentral Gyrus | R | 4 | -36 | 74 | 5.01 | 1113 |
| MTG | R | 50 | -4 | -28 | 5.15 | 1092 |
| Crus I | L | -40 | -62 | -40 | 5.33 | 688 |
| Postcentral Gyrus | L | -42 | -20 | 38 | 5.62 | 530 |
| Caudate | R | 14 | 6 | 20 | 4.77 | 530 |
| Precentral Gyrus | R | 52 | -6 | 46 | 4.93 | 407 |
| Frontal Pole | L | -40 | 52 | -10 | 5.09 | 399 |
| Lingual Gyrus | R | 14 | -70 | -6 | 4.71 | 377 |
| Lingual Gyrus | L | -18 | -72 | -10 | 5.06 | 292 |
| Cuneual Cortex | L | -10 | -78 | 24 | 6.28 | 234 |
| Accumbens | L | -12 | 18 | -10 | 4.94 | 228 |
| Crus I | R | 48 | -64 | -30 | 5.22 | 201 |
| Temporal Pole | R | 54 | 6 | -32 | 4.11 | 107 |
| Crus I | R | 38 | -60 | -38 | 3.97 | 107 |
| LOC | R | 48 | -74 | 8 | 3.99 | 99 |
| ***Recall > Downward CFT*** | | | | | | |
| LOC | R | 30 | -64 | 40 | 7.04 | 12073 |
| *PCC* | L | -6 | -44 | 6 | 6.26 |  |
| *SPL* | L | -30 | -50 | 40 | 6.24 |  |
| *AG/LOC* | L | -36 | -58 | 40 | 6.12 |  |
| *PCC* | L | -2 | -32 | 34 | 6.03 |  |
| *PCC* | R | 2 | -36 | 34 | 6.02 |  |
| MFG | L | -36 | 4 | 34 | 4.99 | 702 |
| vmPFC | L | -6 | 46 | -10 | 5.69 | 647 |
| MFG | R | 40 | 20 | 26 | 4.29 | 563 |
| Precentral Gyrus | R | 38 | -20 | 60 | 4.29 | 189 |
| Fusiform Cortex | R | 42 | -60 | -16 | 4.89 | 174 |
| Postcentral Gyrus | R | 48 | -26 | 54 | 4.03 | 158 |
| Parahippocampal Gyrus | R | 30 | -26 | -20 | 4.10 | 118 |
| ***Recall > Upward CFT*** | | | | | | |
| Hippocampus | L | -34 | -28 | -4 | 6.00 | 7332 |
| *Occipital Pole* | L | -16 | -96 | -10 | 5.96 |  |
| *Occipital Pole* | R | 14 | -90 | 2 | 5.79 |  |
| *Occipital Pole* | L | -14 | -98 | -2 | 5.77 |  |
| *PCC* | L | -4 | -44 | 6 | 5.60 |  |
| *Occipital Pole* | L | -20 | -98 | 0 | 5.53 |  |
| Postcentral Gyrus | L | -40 | -34 | 42 | 5.63 | 1763 |
| IFG | L | -42 | 26 | 22 | 5.54 | 1055 |
| LOC | R | 30 | -64 | 40 | 5.80 | 391 |
| ITG | R | 46 | -52 | -6 | 5.19 | 204 |
| vmPFC | L | -4 | 48 | -10 | 5.02 | 159 |
| Intracalcarine Cortex | L | -20 | -72 | 10 | 4.05 | 158 |
| MFG | R | 56 | 18 | 36 | 3.70 | 115 |
| Fusiform Cortex | R | 32 | -42 | -20 | 3.58 | 100 |
| *Note.* Regions were identified using the Harvard-Oxford Cortical and Subcortical Atlases, and FSL’s Cerebellum Atlas. *MFG*, Middle Frontal Gyrus; *SFG*, Superior Frontal Gyrus; *AG*, Angular Gyrus; *ITG*, Inferior Temporal Gyrus; *OFC*, Orbital Frontal Cortex; *IFG*, Inferior Frontal Gyrus; *LOC*, Lateral Occipital Cortex; *MTG*, Middle Temporal Gyrus; *PCC*, Posterior Cingulate Cortex; *SPL*, Superior Parietal Lobule; *vmPFC*, Ventromedial Prefrontal Cortex. | | | | | | |

### Table S2. Regions of interest for multi-voxel neural pattern similarity analysis (from the Automated Anatomical Labelling atlas 3).

| **Index** | **Name in the atlas** | **Abbreviation** | **Hemi** | **Size in voxels** | **Overlap ratio** |
| --- | --- | --- | --- | --- | --- |
| 3 | Superior frontal gyrus | SFG | L | 4870 | 0.32 |
| 4 | Superior frontal gyrus | SFG | R | 5126 | 0.48 |
| 5 | Middle frontal gyrus | MFG | L | 4507 | 0.35 |
| 6 | Middle frontal gyrus | MFG | R | 4860 | 0.39 |
| 7 | Inferior frontal gyrus, pars opercularis | opercular IFG | L | 1038 | 0.38 |
| 9 | Inferior frontal gyrus, pars triangularis | triangular IFG | L | 2529 | 0.31 |
| 11 | Inferior frontal gyrus, pars orbitalis | orbital IFG | L | 814 | 0.36 |
| 12 | Inferior frontal gyrus, pars orbitalis | orbital IFG | R | 874 | 0.27 |
| 15 | Supplementary motor area | SMA | L | 2147 | 0.31 |
| 19 | Superior frontal gyrus, medial | medial SFG | L | 2992 | 0.32 |
| 20 | Superior frontal gyrus, medial | medial SFG | R | 2134 | 0.39 |
| 21 | Superior frontal gyrus, medial orbital | vmPFC | L | 719 | 0.47 |
| 22 | Superior frontal gyrus, medial orbital | vmPFC | R | 856 | 0.28 |
| 31 | Lateral orbital gyrus | lateral OFC | L | 197 | 0.49 |
| 32 | Lateral orbital gyrus | lateral OFC | R | 188 | 0.26 |
| 39 | Posterior cingulate gyrus | PCC | L | 463 | 0.67 |
| 40 | Posterior cingulate gyrus | PCC | R | 335 | 0.38 |
| 41 | Hippocampus | Hipp | L | 932 | 0.51 |
| 42 | Hippocampus | Hipp | R | 946 | 0.34 |
| 45 | Amygdala | Amyg | L | 220 | 0.33 |
| 47 | Calcarine fissure and surrounding cortex | Cal | L | 2258 | 0.43 |
| 48 | Calcarine fissure and surrounding cortex | Cal | R | 1861 | 0.30 |
| 49 | Cuneus | Cun | L | 1521 | 0.38 |
| 51 | Lingual gyrus | LG | L | 2095 | 0.45 |
| 52 | Lingual gyrus | LG | R | 2300 | 0.44 |
| 53 | Superior occipital gyrus | SOG | L | 1366 | 0.31 |
| 55 | Middle occipital gyrus | MOG | L | 3265 | 0.33 |
| 57 | Inferior occipital gyrus | IOG | L | 941 | 0.55 |
| 58 | Inferior occipital gyrus | IOG | R | 989 | 0.31 |
| 61 | Postcentral gyrus | PoCG | L | 3892 | 0.26 |
| 65 | Inferior parietal gyrus | IPG | L | 2447 | 0.61 |
| 66 | Inferior parietal gyrus | IPG | R | 1345 | 0.46 |
| 67 | SupraMarginal gyrus | SMG | L | 1256 | 0.34 |
| 68 | SupraMarginal gyrus | SMG | R | 1974 | 0.28 |
| 69 | Angular gyrus | AG | L | 1173 | 0.64 |
| 70 | Angular gyrus | AG | R | 1752 | 0.69 |
| 71 | Precuneus | Pcun | L | 3528 | 0.35 |
| 72 | Precuneus | Pcun | R | 3265 | 0.39 |
| 76 | Caudate nucleus | Cau | R | 861 | 0.25 |
| 85 | Superior temporal gyrus | STG | L | 2296 | 0.26 |
| 86 | Superior temporal gyrus | STG | R | 3141 | 0.26 |
| 89 | Middle temporal gyrus | MTG | L | 4942 | 0.27 |
| 90 | Middle temporal gyrus | MTG | R | 4409 | 0.27 |
| 95 | Crus I of cerebellar hemisphere | CERCRU1 | L | 2603 | 0.37 |
| 96 | Crus I of cerebellar hemisphere | CERCRU1 | R | 2648 | 0.32 |
| 101 | Lobule IV, V of cerebellar hemisphere | CER4_5 | L | 1125 | 0.29 |
| 104 | Lobule VI of cerebellar hemisphere | CER6 | R | 1795 | 0.30 |
| 115 | Lobule IV, V of vermis | VER4_5 | LR | 665 | 0.26 |
| 116 | Lobule VI of vermis | VER6 | LR | 371 | 0.56 |
| 119 | Lobule IX of vermis | VER9 | LR | 174 | 0.47 |
| 120 | Lobule X of vermis | VER10 | LR | 112 | 0.27 |
| 152 | Anterior cingulate cortex, subgenual | subgenual ACC | R | 132 | 0.26 |
| 153 | Anterior cingulate cortex, pregenual | pregenual ACC | L | 627 | 0.31 |
| 154 | Anterior cingulate cortex, pregenual | pregenual ACC | R | 648 | 0.50 |
| 157 | Nucleus accumbens | NAcc | L | 163 | 0.30 |
| 158 | Nucleus accumbens | NAcc | R | 143 | 0.27 |

### Table S3. Significant interactions between trait anxiety and counterfactual simulation condition predicting Recall-CFT similarity shift index.

| **Region of interest** | **Effect** | **b** | **SE** | **df** | **t** | **P_FDR_** | **Sig.** |
| --- | --- | --- | --- | --- | --- | --- | --- |
| 12  Right orbital inferior frontal gyrus (IFG) | Intercept | 0.055 | 0.023 | 46.7 | 2.38 | 0.079 |  |
|  | CFT (upward - downward) | -0.013 | 0.024 | 1004.4 | -0.54 | 0.967 |  |
|  | Trait Anxiety | -0.003 | 0.022 | 37.3 | -0.13 | 0.976 |  |
|  | Baseline Arousal | 0.004 | 0.013 | 943.1 | 0.34 | 0.983 |  |
|  | **CFT × Anxiety** | **0.072** | **0.024** | **1006.7** | **3.04** | **0.039** | ***** |
|  | CFT × Arousal | -0.019 | 0.024 | 1012.8 | -0.78 | 0.931 |  |
|  | Anxiety × Arousal | 0.005 | 0.013 | 853.4 | 0.36 | 0.988 |  |
|  | CFT × Anxiety × Arousal | 0.006 | 0.023 | 1014.4 | 0.26 | 0.912 |  |
| 20  Right medial superior frontal gyrus (SFG) | Intercept | 0.016 | 0.016 | 50.8 | 0.98 | 0.544 |  |
|  | CFT (upward - downward) | 0.004 | 0.019 | 1005.4 | 0.20 | 0.967 |  |
|  | Trait Anxiety | -0.017 | 0.015 | 37.6 | -1.14 | 0.976 |  |
|  | Baseline Arousal | 0.000 | 0.010 | 837.9 | -0.02 | 0.983 |  |
|  | **CFT × Anxiety** | **0.065** | **0.019** | **1008.3** | **3.46** | **0.024** | ***** |
|  | CFT × Arousal | -0.025 | 0.019 | 1016.5 | -1.31 | 0.931 |  |
|  | Anxiety × Arousal | -0.006 | 0.010 | 714.6 | -0.56 | 0.988 |  |
|  | CFT × Anxiety × Arousal | -0.003 | 0.018 | 1018.5 | -0.16 | 0.919 |  |
| 21  Left ventromedial prefrontal cortex (vmPFC) | **Intercept** | **0.107** | **0.024** | **50.7** | **4.56** | **0.002** | ****** |
|  | CFT (upward - downward) | 0.019 | 0.027 | 1005.7 | 0.72 | 0.967 |  |
|  | Trait Anxiety | 0.027 | 0.022 | 38.1 | 1.25 | 0.976 |  |
|  | Baseline Arousal | 0.001 | 0.015 | 862.7 | 0.09 | 0.983 |  |
|  | **CFT × Anxiety** | **0.076** | **0.027** | **1008.5** | **2.79** | **0.050** |  |
|  | CFT × Arousal | -0.021 | 0.027 | 1016.1 | -0.76 | 0.931 |  |
|  | Anxiety × Arousal | -0.008 | 0.015 | 745.3 | -0.55 | 0.988 |  |
|  | CFT × Anxiety × Arousal | -0.002 | 0.026 | 1018.1 | -0.09 | 0.951 |  |
| 22  Right ventromedial prefrontal cortex (vmPFC) | **Intercept** | **0.083** | **0.023** | **50.7** | **3.52** | **0.010** | ***** |
|  | CFT (upward - downward) | 0.012 | 0.027 | 1005.5 | 0.46 | 0.967 |  |
|  | Trait Anxiety | 0.007 | 0.022 | 37.8 | 0.32 | 0.976 |  |
|  | Baseline Arousal | 0.004 | 0.015 | 852.8 | 0.28 | 0.983 |  |
|  | CFT × Anxiety | 0.048 | 0.027 | 1008.4 | 1.76 | 0.148 |  |
|  | CFT × Arousal | -0.014 | 0.027 | 1016.2 | -0.51 | 0.998 |  |
|  | Anxiety × Arousal | 0.003 | 0.015 | 732.8 | 0.20 | 0.988 |  |
|  | CFT × Anxiety × Arousal | 0.023 | 0.026 | 1018.2 | 0.86 | 0.861 |  |
| 31  Left lateral orbital frontal cortex (OFC) | **Intercept** | **0.109** | **0.030** | **49.7** | **3.59** | **0.010** | ***** |
|  | CFT (upward - downward) | -0.032 | 0.035 | 1005.0 | -0.93 | 0.967 |  |
|  | Trait Anxiety | -0.020 | 0.028 | 37.3 | -0.71 | 0.976 |  |
|  | Baseline Arousal | 0.013 | 0.019 | 857.3 | 0.68 | 0.983 |  |
|  | CFT × Anxiety | 0.078 | 0.035 | 1007.8 | 2.24 | 0.074 |  |
|  | CFT × Arousal | -0.028 | 0.035 | 1015.7 | -0.81 | 0.931 |  |
|  | Anxiety × Arousal | 0.004 | 0.019 | 737.9 | 0.22 | 0.988 |  |
|  | CFT × Anxiety × Arousal | -0.032 | 0.034 | 1017.7 | -0.95 | 0.861 |  |
| 32  Right lateral orbital frontal cortex (OFC) | **Intercept** | **0.105** | **0.026** | **54.1** | **4.09** | **0.004** | ****** |
|  | CFT (upward - downward) | 0.021 | 0.034 | 1005.4 | 0.62 | 0.967 |  |
|  | Trait Anxiety | -0.006 | 0.023 | 36.8 | -0.25 | 0.976 |  |
|  | Baseline Arousal | 0.008 | 0.018 | 715.5 | 0.44 | 0.983 |  |
|  | CFT × Anxiety | 0.068 | 0.034 | 1009.1 | 2.02 | 0.111 |  |
|  | CFT × Arousal | -0.035 | 0.034 | 1019.6 | -1.02 | 0.931 |  |
|  | Anxiety × Arousal | -0.002 | 0.018 | 580.1 | -0.10 | 0.988 |  |
|  | CFT × Anxiety × Arousal | -0.008 | 0.033 | 1022.1 | -0.24 | 0.912 |  |
| 40  Right posterior cingulate cortex (PCC) | Intercept | -0.016 | 0.017 | 57.4 | -0.96 | 0.545 |  |
|  | CFT (upward - downward) | -0.023 | 0.023 | 1007.1 | -1.00 | 0.967 |  |
|  | Trait Anxiety | 0.028 | 0.015 | 38.4 | 1.87 | 0.976 |  |
|  | Baseline Arousal | -0.002 | 0.012 | 702.4 | -0.20 | 0.983 |  |
|  | **CFT × Anxiety** | **0.066** | **0.023** | **1010.7** | **2.93** | **0.039** | ***** |
|  | CFT × Arousal | -0.026 | 0.023 | 1021.2 | -1.14 | 0.931 |  |
|  | Anxiety × Arousal | 0.005 | 0.012 | 568.5 | 0.43 | 0.988 |  |
|  | CFT × Anxiety × Arousal | -0.024 | 0.022 | 1023.6 | -1.08 | 0.861 |  |
| 67  Left supramarginal gyrus (SMG) | **Intercept** | **-0.048** | **0.015** | **57.7** | **-3.12** | **0.023** | ***** |
|  | CFT (upward - downward) | 0.021 | 0.021 | 1006.7 | 0.99 | 0.967 |  |
|  | Trait Anxiety | 0.010 | 0.014 | 37.8 | 0.72 | 0.976 |  |
|  | Baseline Arousal | -0.007 | 0.011 | 671.8 | -0.63 | 0.983 |  |
|  | CFT × Anxiety | 0.015 | 0.021 | 1010.6 | 0.73 | 0.503 |  |
|  | CFT × Arousal | 0.003 | 0.021 | 1021.7 | 0.12 | 0.998 |  |
|  | Anxiety × Arousal | 0.010 | 0.011 | 538.3 | 0.88 | 0.988 |  |
|  | CFT × Anxiety × Arousal | -0.011 | 0.020 | 1024.2 | -0.56 | 0.912 |  |
| 69  Left angular gyrus (AG) | **Intercept** | **-0.045** | **0.014** | **59.1** | **-3.12** | **0.023** | ***** |
|  | CFT (upward - downward) | 0.029 | 0.020 | 1006.6 | 1.42 | 0.967 |  |
|  | Trait Anxiety | 0.011 | 0.013 | 37.4 | 0.86 | 0.976 |  |
|  | Baseline Arousal | -0.004 | 0.011 | 622.0 | -0.39 | 0.983 |  |
|  | CFT × Anxiety | 0.026 | 0.020 | 1010.8 | 1.27 | 0.271 |  |
|  | CFT × Arousal | -0.003 | 0.020 | 1023.0 | -0.16 | 0.998 |  |
|  | Anxiety × Arousal | -0.008 | 0.010 | 491.7 | -0.80 | 0.988 |  |
|  | CFT × Anxiety × Arousal | -0.017 | 0.020 | 1025.6 | -0.87 | 0.861 |  |
| 70  Right angular gyrus (AG) | **Intercept** | **-0.059** | **0.016** | **52.8** | **-3.77** | **0.008** | ****** |
|  | CFT (upward - downward) | 0.014 | 0.019 | 1005.8 | 0.74 | 0.967 |  |
|  | Trait Anxiety | -0.001 | 0.014 | 37.7 | -0.07 | 0.976 |  |
|  | Baseline Arousal | 0.002 | 0.010 | 789.3 | 0.15 | 0.983 |  |
|  | CFT × Anxiety | 0.038 | 0.019 | 1009.1 | 1.97 | 0.114 |  |
|  | CFT × Arousal | -0.013 | 0.019 | 1018.1 | -0.66 | 0.943 |  |
|  | Anxiety × Arousal | 0.003 | 0.010 | 658.8 | 0.26 | 0.988 |  |
|  | CFT × Anxiety × Arousal | -0.015 | 0.019 | 1020.3 | -0.82 | 0.861 |  |
| 101  Left Lobule IV, V of cerebellar hemisphere (CER4_5) | Intercept | 0.005 | 0.016 | 52.4 | 0.30 | 0.870 |  |
|  | CFT (upward - downward) | -0.006 | 0.019 | 1006.3 | -0.29 | 0.967 |  |
|  | Trait Anxiety | -0.007 | 0.015 | 38.6 | -0.47 | 0.976 |  |
|  | Baseline Arousal | -0.011 | 0.011 | 839.3 | -1.07 | 0.983 |  |
|  | **CFT × Anxiety** | **0.058** | **0.020** | **1009.2** | **2.99** | **0.039** | ***** |
|  | CFT × Arousal | 0.007 | 0.020 | 1017.2 | 0.34 | 0.998 |  |
|  | Anxiety × Arousal | -0.018 | 0.010 | 717.0 | -1.74 | 0.988 |  |
|  | CFT × Anxiety × Arousal | -0.025 | 0.019 | 1019.2 | -1.30 | 0.861 |  |
| 104  Right Lobule VI of cerebellar hemisphere (CER6) | Intercept | 0.044 | 0.019 | 47.5 | 2.36 | 0.079 |  |
|  | CFT (upward - downward) | -0.005 | 0.019 | 1005.3 | -0.26 | 0.967 |  |
|  | Trait Anxiety | -0.005 | 0.017 | 38.3 | -0.27 | 0.976 |  |
|  | Baseline Arousal | 0.000 | 0.010 | 954.2 | 0.04 | 0.983 |  |
|  | **CFT × Anxiety** | **0.062** | **0.019** | **1007.4** | **3.34** | **0.024** | ***** |
|  | CFT × Arousal | 0.000 | 0.019 | 1013.1 | -0.01 | 0.998 |  |
|  | Anxiety × Arousal | -0.005 | 0.010 | 870.8 | -0.45 | 0.988 |  |
|  | CFT × Anxiety × Arousal | -0.027 | 0.018 | 1014.7 | -1.51 | 0.861 |  |
| *Note*. This table shows fitted model results for ROIs who’s main and interaction effects of interest (emphasized in bold) were significant after FDR correction for all 56 tested regions (****p* < 0.001, ***p* < 0.01, **p* < 0.05). All continuous variables were mean-centered and standardized. | | | | | | | |

### Table S4. Significant interactions between trait anxiety and counterfactual simulation condition predicting CFT-CFT similarity index.

| **Region of interest** | **Effect** | **b** | **SE** | **df** | **t** | **P_FDR_** | **Sig.** |
| --- | --- | --- | --- | --- | --- | --- | --- |
| 3  Left superior frontal gyrus (SFG) | Intercept | 0.026 | 0.004 | 37.3 | 6.78 | 0.000 | *** |
|  | CFT (upward - downward) | 0.003 | 0.003 | 1012.2 | 1.00 | 0.722 |  |
|  | Trait Anxiety | 0.005 | 0.004 | 37.7 | 1.22 | 0.659 |  |
|  | Baseline Arousal | 0.000 | 0.002 | 1024.4 | 0.15 | 0.950 |  |
|  | **CFT × Anxiety** | **0.009** | **0.003** | **1013.4** | **2.66** | **0.048** | ***** |
|  | CFT × Arousal | -0.004 | 0.003 | 1018.0 | -1.33 | 0.639 |  |
|  | Anxiety × Arousal | 0.000 | 0.002 | 983.2 | -0.25 | 0.991 |  |
|  | CFT × Anxiety × Arousal | 0.002 | 0.003 | 1018.5 | 0.52 | 0.975 |  |
| 15  Left supplementary motor area (SMA) | Intercept | 0.023 | 0.004 | 37.1 | 5.62 | 0.000 | *** |
|  | CFT (upward - downward) | -0.003 | 0.003 | 1011.8 | -0.78 | 0.722 |  |
|  | Trait Anxiety | 0.005 | 0.004 | 37.5 | 1.33 | 0.592 |  |
|  | Baseline Arousal | -0.001 | 0.002 | 1032.7 | -0.28 | 0.950 |  |
|  | **CFT × Anxiety** | **0.009** | **0.003** | **1012.9** | **2.62** | **0.048** | ***** |
|  | CFT × Arousal | -0.002 | 0.003 | 1017.1 | -0.53 | 0.847 |  |
|  | Anxiety × Arousal | 0.001 | 0.002 | 1000.7 | 0.39 | 0.991 |  |
|  | CFT × Anxiety × Arousal | 0.006 | 0.003 | 1017.6 | 1.97 | 0.584 |  |
| 19  Left medial superior frontal gyrus (SFG) | Intercept | 0.030 | 0.004 | 36.6 | 7.04 | 0.000 | *** |
|  | CFT (upward - downward) | 0.001 | 0.004 | 1012.0 | 0.32 | 0.913 |  |
|  | Trait Anxiety | 0.008 | 0.004 | 37.2 | 1.93 | 0.341 |  |
|  | Baseline Arousal | 0.002 | 0.002 | 999.6 | 0.82 | 0.950 |  |
|  | **CFT × Anxiety** | **0.013** | **0.004** | **1013.4** | **3.15** | **0.019** | ***** |
|  | CFT × Arousal | -0.003 | 0.004 | 1019.0 | -0.77 | 0.847 |  |
|  | Anxiety × Arousal | 0.001 | 0.002 | 936.7 | 0.49 | 0.991 |  |
|  | CFT × Anxiety × Arousal | 0.002 | 0.004 | 1019.7 | 0.44 | 0.975 |  |
| 20  Right medial superior frontal gyrus (SFG) | Intercept | 0.027 | 0.004 | 37.3 | 7.04 | 0.000 | *** |
|  | CFT (upward - downward) | 0.007 | 0.004 | 1012.6 | 1.90 | 0.383 |  |
|  | Trait Anxiety | 0.008 | 0.004 | 37.8 | 2.06 | 0.341 |  |
|  | Baseline Arousal | 0.001 | 0.002 | 996.7 | 0.26 | 0.950 |  |
|  | **CFT × Anxiety** | **0.011** | **0.004** | **1014.1** | **3.04** | **0.022** | ***** |
|  | CFT × Arousal | -0.005 | 0.004 | 1019.7 | -1.34 | 0.639 |  |
|  | Anxiety × Arousal | 0.001 | 0.002 | 931.9 | 0.26 | 0.991 |  |
|  | CFT × Anxiety × Arousal | 0.000 | 0.004 | 1020.4 | 0.09 | 0.988 |  |
| 39  Left posterior cingulate cortex (PCC) | Intercept | 0.023 | 0.005 | 36.8 | 4.82 | 0.000 | *** |
|  | CFT (upward - downward) | 0.015 | 0.005 | 1012.4 | 2.96 | 0.179 |  |
|  | Trait Anxiety | -0.002 | 0.005 | 37.4 | -0.39 | 0.903 |  |
|  | Baseline Arousal | 0.002 | 0.003 | 976.7 | 0.84 | 0.950 |  |
|  | **CFT × Anxiety** | **0.022** | **0.005** | **1014.0** | **4.42** | **0.001** | ****** |
|  | CFT × Arousal | -0.009 | 0.005 | 1020.3 | -1.74 | 0.528 |  |
|  | Anxiety × Arousal | 0.002 | 0.003 | 898.5 | 0.58 | 0.991 |  |
|  | CFT × Anxiety × Arousal | -0.005 | 0.005 | 1021.1 | -1.01 | 0.975 |  |
| 67  Left supramarginal gyrus (SMG) | Intercept | 0.026 | 0.004 | 36.5 | 6.79 | 0.000 | *** |
|  | CFT (upward - downward) | 0.003 | 0.004 | 1012.9 | 0.57 | 0.817 |  |
|  | Trait Anxiety | 0.002 | 0.004 | 37.2 | 0.48 | 0.903 |  |
|  | Baseline Arousal | -0.002 | 0.002 | 906.4 | -0.73 | 0.950 |  |
|  | **CFT × Anxiety** | **0.013** | **0.005** | **1014.9** | **2.79** | **0.042** | ***** |
|  | CFT × Arousal | 0.000 | 0.005 | 1023.0 | 0.02 | 0.995 |  |
|  | Anxiety × Arousal | 0.002 | 0.002 | 795.6 | 0.77 | 0.991 |  |
|  | CFT × Anxiety × Arousal | -0.002 | 0.004 | 1023.9 | -0.36 | 0.975 |  |
| 69  Left angular gyrus (AG) | Intercept | 0.028 | 0.005 | 36.6 | 5.49 | 0.000 | *** |
|  | CFT (upward - downward) | 0.010 | 0.004 | 1011.7 | 2.15 | 0.299 |  |
|  | Trait Anxiety | 0.001 | 0.005 | 37.1 | 0.16 | 0.967 |  |
|  | Baseline Arousal | -0.001 | 0.003 | 1016.8 | -0.45 | 0.950 |  |
|  | **CFT × Anxiety** | **0.015** | **0.005** | **1013.0** | **3.41** | **0.013** | ***** |
|  | CFT × Arousal | -0.003 | 0.005 | 1018.0 | -0.77 | 0.847 |  |
|  | Anxiety × Arousal | 0.002 | 0.003 | 968.0 | 0.75 | 0.991 |  |
|  | CFT × Anxiety × Arousal | -0.007 | 0.004 | 1018.6 | -1.55 | 0.762 |  |
| 70  Right angular gyrus (AG) | Intercept | 0.023 | 0.005 | 36.8 | 4.98 | 0.000 | *** |
|  | CFT (upward - downward) | 0.007 | 0.004 | 1012.0 | 1.62 | 0.430 |  |
|  | Trait Anxiety | 0.009 | 0.005 | 37.3 | 1.84 | 0.341 |  |
|  | Baseline Arousal | -0.003 | 0.002 | 1002.4 | -1.13 | 0.950 |  |
|  | **CFT × Anxiety** | **0.012** | **0.004** | **1013.5** | **2.60** | **0.048** | ***** |
|  | CFT × Arousal | -0.008 | 0.004 | 1019.0 | -1.72 | 0.528 |  |
|  | Anxiety × Arousal | 0.000 | 0.002 | 941.7 | -0.10 | 0.991 |  |
|  | CFT × Anxiety × Arousal | 0.001 | 0.004 | 1019.7 | 0.17 | 0.988 |  |
| 76  Right caudate nucleus | Intercept | 0.019 | 0.003 | 35.3 | 5.77 | 0.000 | *** |
|  | CFT (upward - downward) | -0.010 | 0.004 | 1011.5 | -2.61 | 0.260 |  |
|  | **Trait Anxiety** | **0.013** | **0.003** | **36.0** | **3.96** | **0.019** | ***** |
|  | Baseline Arousal | 0.003 | 0.002 | 921.5 | 1.58 | 0.950 |  |
|  | CFT × Anxiety | 0.006 | 0.004 | 1013.5 | 1.59 | 0.266 |  |
|  | CFT × Arousal | 0.009 | 0.004 | 1021.4 | 2.31 | 0.528 |  |
|  | Anxiety × Arousal | 0.002 | 0.002 | 815.2 | 0.79 | 0.991 |  |
|  | CFT × Anxiety × Arousal | 0.002 | 0.004 | 1022.3 | 0.48 | 0.975 |  |
| 89  Left middle temporal gyrus (MTG) | Intercept | 0.027 | 0.004 | 36.9 | 7.24 | 0.000 | *** |
|  | CFT (upward - downward) | 0.005 | 0.003 | 1011.6 | 1.53 | 0.468 |  |
|  | Trait Anxiety | 0.004 | 0.004 | 37.3 | 1.05 | 0.767 |  |
|  | Baseline Arousal | -0.001 | 0.002 | 1035.0 | -0.74 | 0.950 |  |
|  | **CFT × Anxiety** | **0.010** | **0.003** | **1012.7** | **3.23** | **0.018** | ***** |
|  | CFT × Arousal | 0.000 | 0.003 | 1016.8 | 0.01 | 0.995 |  |
|  | Anxiety × Arousal | 0.002 | 0.002 | 1005.7 | 0.96 | 0.991 |  |
|  | CFT × Anxiety × Arousal | 0.006 | 0.003 | 1017.3 | 1.94 | 0.584 |  |
| 90  Right middle temporal gyrus (MTG) | Intercept | 0.024 | 0.004 | 37.1 | 6.31 | 0.000 | *** |
|  | CFT (upward - downward) | -0.001 | 0.003 | 1011.6 | -0.18 | 0.944 |  |
|  | Trait Anxiety | 0.007 | 0.004 | 37.4 | 1.77 | 0.341 |  |
|  | Baseline Arousal | -0.001 | 0.002 | 1038.9 | -0.62 | 0.950 |  |
|  | **CFT × Anxiety** | **0.012** | **0.003** | **1012.7** | **4.14** | **0.001** | ****** |
|  | CFT × Arousal | 0.001 | 0.003 | 1016.4 | 0.24 | 0.932 |  |
|  | Anxiety × Arousal | 0.001 | 0.002 | 1015.1 | 0.71 | 0.991 |  |
|  | CFT × Anxiety × Arousal | 0.005 | 0.003 | 1016.9 | 1.63 | 0.727 |  |
| 95  Left crus I of cerebellar hemisphere  (CERCRU1) | Intercept | 0.018 | 0.003 | 35.8 | 5.91 | 0.000 | *** |
|  | CFT (upward - downward) | 0.000 | 0.003 | 1011.8 | -0.02 | 0.982 |  |
|  | Trait Anxiety | 0.003 | 0.003 | 36.4 | 0.91 | 0.767 |  |
|  | Baseline Arousal | -0.004 | 0.002 | 940.3 | -1.97 | 0.950 |  |
|  | **CFT × Anxiety** | **0.009** | **0.003** | **1013.7** | **2.76** | **0.042** | ***** |
|  | CFT × Arousal | -0.004 | 0.003 | 1021.1 | -1.15 | 0.690 |  |
|  | Anxiety × Arousal | -0.002 | 0.002 | 842.4 | -0.85 | 0.991 |  |
|  | CFT × Anxiety × Arousal | -0.002 | 0.003 | 1022.0 | -0.47 | 0.975 |  |
| *Note.* This table shows fitted model results for ROIs who’s main and interaction effects of interest (emphasized in bold) were significant after FDR correction for all 56 tested regions (****p* < 0.001, ***p* < 0.01, **p* < 0.05). All continuous variables were mean-centered and standardized. | | | | | | | |
